# Fault Diagnosis in Chemical Reactors with Data-Driven Methods

**DOI:** 10.1021/acs.iecr.4c04042

**Published:** 2025-03-08

**Authors:** Pu Du, Nabil M. Abdel Jabbar, Benjamin A. Wilhite, Costas Kravaris

**Affiliations:** † Artie McFerrin Department of Chemical Engineering, Texas A&M University, College Station, Texas 77843, United States of America; ‡ Chemical and Biological Engineering Department, American University of Sharjah, Sharjah 26666, United Arab Emirates

## Abstract

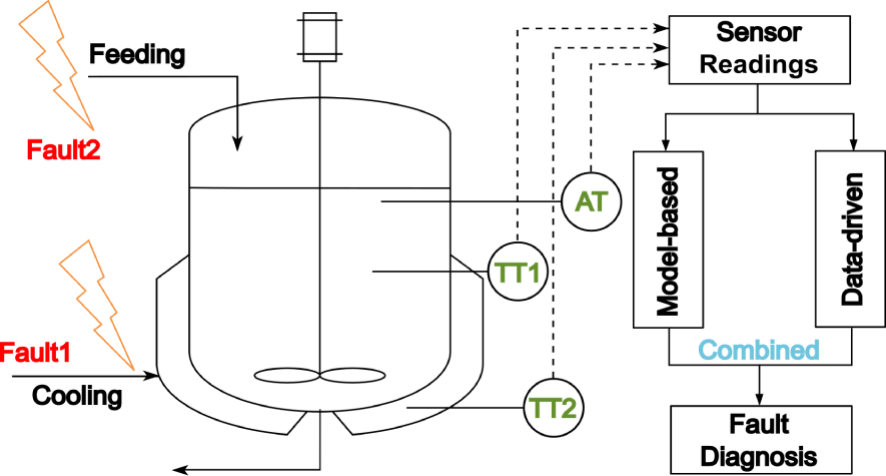

This study investigates fault diagnosis, encompassing fault detection, isolation, and estimation, with experimental data in a continuous stirred-tank reactor (CSTR) for the liquid-phase catalytic oxidation of 3-picoline with hydrogen peroxide. Two key faults were examined: coolant inlet temperature spikes (fault 1) and 3-picoline feed concentration decreases (fault 2). Data-driven methods, including random forest (RF) and *k*-nearest neighbors (KNN), successfully detected, isolated, and estimated faults under nominal conditions. However, both data-driven and model-based residual generators were disrupted by a shift in the heat transfer coefficient (*U*). An isolation forest (IF) algorithm was used for anomaly detection and model recalibration, restoring model-based performance. Updated data sets enabled RF and KNN to adapt effectively, demonstrating their scalability and adaptability. Experimental results highlight the strengths of both methods, advocating for a combined framework for robust fault diagnosis.

## 1 Introduction

Fault diagnosis is a critical aspect of process safety in the chemical industry, where the reliability of operations can directly influence productivity, economic performance, and, most importantly, safety.^1,2^ Chemical processes are highly complex and involve multiple interrelated variables, often operating under extreme conditions of temperature, pressure, and chemical reactivity. Any undetected faults within these systems can lead to equipment malfunction, production losses, environmental hazards, and, in severe cases, catastrophic accidents involving the loss of life. Therefore, ensuring robust and timely fault diagnosis systems is paramount to mitigate risks and enhance the overall reliability of industrial operations.^3^

Fault diagnosis systems aim to detect, isolate, and estimate faults as early as possible to enable timely corrective actions, with each objective becoming progressively more challenging. In the chemical industry, faults can arise from various sources including sensor failures, actuator malfunctions, equipment degradation, and process disturbances. As processes become more automated and large-scale, human operators often face challenges in manually identifying abnormal conditions due to the overwhelming amount of data generated by process sensors. Hence, automated fault diagnosis methods have gained prominence as key components of process safety frameworks. Early and accurate diagnosis not only prevents accidents but also optimizes maintenance schedules, reduces downtime, and enhances process efficiency. The necessity of robust fault diagnosis systems in the chemical industry has propelled the development of two primary approaches: model-based and data-driven methods.^4−6^

Each approach offers distinct advantages and faces its own set of challenges. Model-based fault diagnosis involves constructing a mathematical representation of the system to compare real-time data with model-predicted outputs. Deviations from the expected behavior, known as residuals, are indicative of faults.^7^ In contrast, data-driven approaches rely on historical operational data to train machine learning models to detect and classify faults.^8,9^ While model-based methods leverage knowledge of the system’s physics, data-driven techniques exploit patterns and trends hidden within large data sets.

Model-based fault diagnosis techniques have long been favored in chemical process control due to their strong foundation in process physics. These methods are based on first principles—such as mass and energy balances—or empirical models that describe the behavior of the system. The core idea is to generate residuals, or error signals, by comparing the system’s actual measurements with predictions from the model. If a fault occurs, the residuals will deviate significantly from zero, indicating abnormal behavior. Popular model-based fault diagnosis techniques include observer-based,^10−13^ parameter estimation,^7,14^ and parity space approaches.^15,16^

Model-based approaches offer the advantage of physical insight, enabling fault detection, root cause analysis, and fault size estimation without relying on extensive historical data. They are particularly reliable for rare or unmonitored faults but are heavily dependent on the accuracy of the system model. Developing such models for complex chemical processes is challenging and time-consuming, requiring a deep knowledge of process dynamics and operational variables. Additionally, real-world systems evolve over time due to factors such as equipment aging or operational changes, leading to model mismatch and potential diagnostic errors. Adaptive modeling is often needed to recalibrate the model, adding complexity and effort, which limits the practical implementation of model-based methods despite their reliability.

On the other hand, data-driven fault diagnosis methods have gained increasing popularity with the rise of machine learning and big data analytics. Data-driven approaches do not require explicit knowledge of the underlying system dynamics.^8,17^ Instead, they rely on patterns, correlations, and anomalies present in the historical process data. Techniques such as random forest^18,19^ (RF), isolation forest^20,21^ (IF), support vector machines^22,23^ (SVM), artificial neural networks^24−26^ (ANN), and principal component analysis^22,27,28^ (PCA) are commonly used in data-driven fault diagnosis applications. John MacGregor and Nomikos have made monumental contributions to process monitoring and fault detection in chemical processes, proposing a PCA-based approach using only data from successful batches to monitor a styrene–butadiene semibatch reactor.^29^ An advanced fault isolation method has been developed to handle both simple and complex faults by extracting fault signatures and comparing them with a fault library of historical data.^30^ Additionally, a fault-tolerant control strategy employing data-driven latent variable models constructed from historical process data is highlighted, emphasizing their reduced dimensionality and interpretability.^31^ Recent studies, particularly in Industrial & Engineering Chemistry Research (I&ECR), have focused on integrating machine learning (ML) and artificial intelligence (AI) with process monitoring and fault diagnosis.^32^ By linking fault diagnosis models with real-time digital replicas of physical systems, these approaches enable proactive maintenance, predictive analytics, and performance optimization.^33−36^

The strength of data-driven methods lies in their ability to handle complex, nonlinear systems without the need for detailed models of the process. They are particularly well-suited for systems in which developing an accurate model is impractical or infeasible. Moreover, once trained, data-driven models can be deployed to monitor systems in real-time and detect a wide range of faults with minimal human intervention. These methods are also highly scalable, making them applicable to large and complex processes with multiple sensors and data points. However, the major challenge associated with data-driven approaches is the requirement for large and diverse data sets. In many cases, particularly for fault size estimation, data-driven models often require training on data sets that encompass a diverse range of fault scenarios to ensure satisfactory performance.^37,38^ In the chemical industry, where processes often run under nominal operating conditions for extended periods, it is difficult to obtain sufficient data representing the various faulty states.

Recently, combined fault diagnosis methods, which incorporate the strengths of model-based and data-driven techniques, have emerged as a promising solution to overcome the limitations of both approaches.^39−41^ These methods aim to integrate the physical insights offered by model-based approaches with the pattern-recognition capabilities of data-driven methods. For instance, model residuals can be used as inputs to a machine learning model, enabling more accurate fault classification. Alternatively, data-driven methods can be used to update model parameters in real-time, improving the model’s adaptability to changing system conditions.

Despite the potential advantages of combined approaches, their practical implementation remains limited, especially when applied to experimental data for achieving all three aspects of fault diagnosis: detection, isolation, and estimation. Most studies in the literature focus on simulation-based validation, where fault scenarios can be artificially generated and tested. For example, the Tennessee Eastman (TE) process is a common testbed for data-driven and model-based methods.^42−44^ As for experimental study, a hybrid approach combining an extended Kalman filter (EKF) with a probabilistic neural network classifier has been successfully applied for fault detection and diagnosis in fed-batch and batch reactors, providing accurate monitoring through the estimation of reactor parameters and classification of fault types.^45^ A robust fault detection methodology for hybrid process systems, incorporating tools from unknown input observer theory and Lyapunov stability, has been developed to reliably distinguish between faults, mode transitions, and uncertainties.^46^ A hybrid data/model-based approach combining SVM with an observer is proposed for fault detection and isolation in nonlinear chemical reactions, effectively reducing the reliance on precise process models or extensive training data.^23^ A hybrid model combining first-principles and neural networks was developed for automatic fault detection and identification, leveraging both simulation data and historical process information. Tested on real data from a methanol–water distillation column, this method outperformed traditional first-principles models by effectively identifying faults and demonstrating its potential for application in refining and petrochemical processes.^47^

Recently, we introduced a comprehensive fault diagnosis methodology for a CSTR chemical reaction system, leveraging model-based residual generators as estimators, systematic data processing to mitigate noise, and predefined thresholds for fault alarms. These residual generators, designed as functional observers decoupled from disturbances, estimate fault sizes. Fault isolation is achieved through multiple independent residual generators.^48,49^ The experiment successfully demonstrated the effectiveness of fault diagnosis in a CSTR across various fault scenarios.^50^ During these experiments, an intriguing phenomenon caught our attention: after switching equipment, the previous model’s performance declined. Further experimentation suggested that this might be due to a change in the heat transfer coefficient. This raised important questions about how to detect model mismatches or parameter changes and how we could leverage known experimental data to enhance the fault diagnosis. Addressing these issues was essential to our work.

In this study, we examine data-driven approaches for fault diagnosis in chemical reactors, comparing them with model-based observers. While model-based residual generators excel in robustness and accuracy, especially when the system’s dynamics are well understood, data-driven methods like RF and KNN offer promising, scalable solutions. Whereas both methods performed well under nominal conditions, system parameter changes, like shifts in the heat transfer coefficient (*U*), posed challenges for both approaches. To address this, we implemented an isolation forest (IF) algorithm for anomaly detection and model recalibration. The study shows that combining data-driven and model-based methods can enhance fault diagnosis, with data-driven techniques becoming more robust after training in updated system conditions.

## 2 Background and Method

This section introduces the reaction system and summarizes key results achieved using model-based fault diagnosis techniques. However, some challenges remain that cannot be effectively addressed by model-based methods alone. This limitation motivates the exploration of a hybrid approach that leverages the strengths of both model- and data-driven techniques. Additionally, the fundamentals of the data-driven methods applied in this work are briefly outlined.

### 2.1 Reactor (CSTR) Model and Experiment Setup

**Figure 1:**
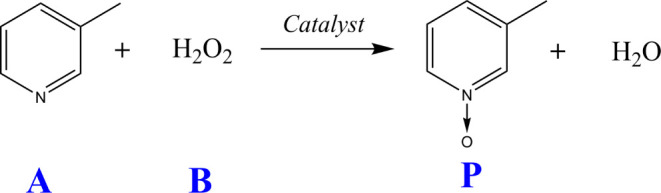
Reaction of catalyzed 3-picoline (A) *N*-oxidation with hydrogen peroxide (B) to produce *N*-oxidized 3-picoline (P) and water.

The *N*-oxidation of alkylpyridine is a crucial reaction in drug synthesis and pharmaceutical applications. Studies have shown that a continuous stirred tank reactor (CSTR) is an effective system for the *N*-oxidation of 3-picoline using hydrogen peroxide,^51^ a process aligned with green chemistry principles. The reaction mechanism is illustrated in Figure 1.

**Figure 2:**
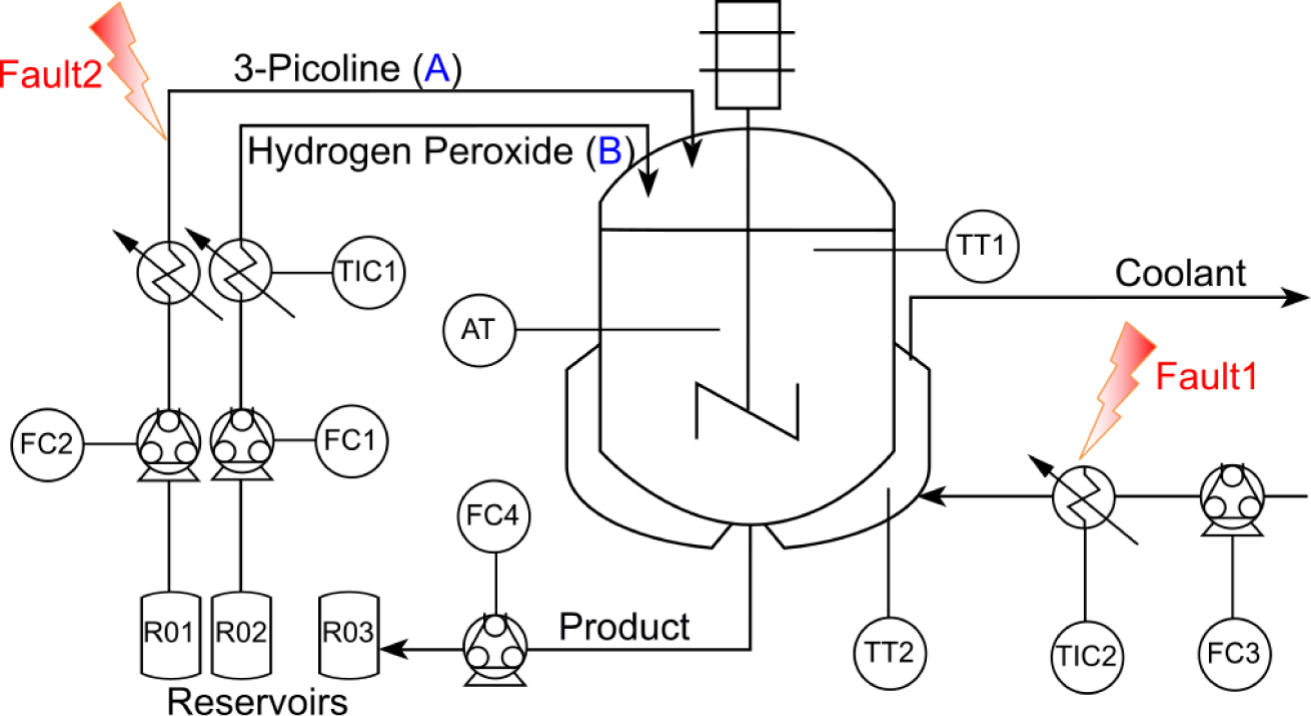
Schematic of experimental apparatus, indicating sensor and control locations.

The CSTR setup and experimental process are schematically represented in Figure 2, with a 50 mL jacketed glass reactor. The objective is to detect, isolate, and estimate two faults in this process (for detailed information, refer to the cited work). During the experiments, faults were introduced, and sensor readings (TT1 for reactor temperature, TT2 for jacket temperature, and AT for 3-picoline concentration) were used to successfully diagnose faults. This was achieved using model-based residual generators derived from the functional observer applied to the system equations (eqs 1–5). Fault detection, isolation, and estimation were successfully achieved in our experiments using model-based residual generators.^50^

The reaction model is built with mass and energy balances, where *C*_*A*_ is the concentration of reactant 3-picoline, *C*_*B*_ is the concentration of reactant hydrogen peroxide, *w*(*t*) is the unknown kinetics variation, *R*(*C*_*A*_,*C*_*B*_,*T*) is the reaction rate, *T* is the reactor temperature, *δ*_*t*_ = 0.1 s as the discretization time interval, and *T*_*j*_ is the jacket temperature. The system dynamics and the specifics of the residual generators are detailed as follows:^50^
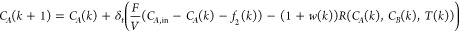
1
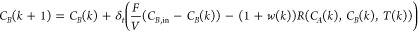
2
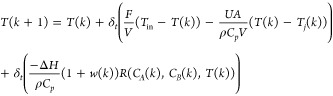
3
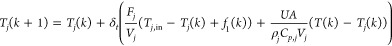
4
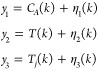
5

Residual generators were constructed for this system to implement fault diagnosis, which remain below a threshold if there is no fault happening and respond to faults (*f*_1_,*f*_2_), and to provide an estimate of these fault sizes.

For fault 1, a spike in coolant feed temperature *T*_*j,*in_, the following functional observer was built,
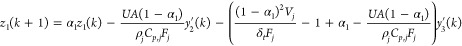
6
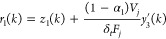
7

Whereas the detection of fault 2, inlet feed ratio of *C*_*A,*in_, and the 3-picoline concentration decrease, similarly, the following functional observer was built.
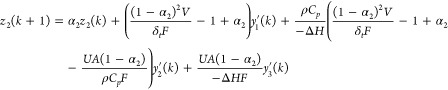
8
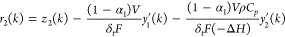
9

In eqs 6–9, , , and  represent the output measurements in deviation form, α_1_ and α_2_ are tunable parameters that represent the observer eigenvalues, *r*_1_ and *r*_2_ are the residuals that also represent estimates of *f*_1_ and *f*_2_, respectively, under the assumption that they are of step or ramp type. The details of individual parameter values are given in Table S1.^50^

It is important to emphasize that the residual generators are unaffected by fluctuations in the reaction kinetic rate, which could otherwise introduce significant errors. Moreover, the two residual generators are decoupled, allowing for effective fault isolation. The experimental results demonstrated successful fault diagnosis for both faults, providing highly accurate estimates of their magnitudes.^50^

### 2.2 Random Forest (RF), *K*-Nearest Neighbors (KNN), and Artificial Neural Networks (ANN) for Fault Diagnosis

The random forest regressor is an ensemble learning technique tailored for regression tasks.^18^ It constructs multiple decision trees during training and averages their predictions to enhance accuracy and mitigate overfitting. Unlike individual decision trees, which are prone to high variance and overfitting, random forest uses bagging (bootstrap aggregation) and random feature selection to build a more robust and generalized model. This approach reduces the likelihood of overfitting and improves the model’s performance on unseen data.

The random forest algorithm applies bootstrap sampling to generate multiple training subsets. Given a data set , where *x*_*i*_ is the input feature and *y*_*i*_ is the target variable, the algorithm generates *t* bootstrap samples. A bootstrap sample *D*_*b*_ is used to train the decision tree. The remaining data, called the out-of-bag (OOB) sample, can be used to estimate the model’s performance.

Each decision tree in the forest is constructed at each node; a subset of features *F*_*m*_ ⊆ *F* is selected at random. The splitting criterion used in regression is typically based on minimizing the mean squared error (MSE). Once the tree is fully grown (or meets a stopping criterion, like maximum depth), it can make predictions for new data. The prediction for a new point *x* is the average of the target values *y* for all samples that fall into the same leaf node as *y*:
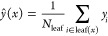
10

After multiple decision trees {*T*_1_,*T*_2_,_···_,*T*_*t*_}, the final prediction for new input *x* is obtained by averaging the predictions of all trees:
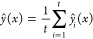
11

Where  is the prediction of the *i*-th tree.

The *k*-nearest neighbors (KNN) regressor is a nonparametric, instance-based learning algorithm used for regression tasks.^52^ Unlike statistical model-based methods, such as random forest, KNN does not require explicit training or model fitting. Instead, it predicts the target value by averaging the target values of the *k*-nearest neighbors in the feature space, relying solely on the stored training data.

KNN uses a distance metric, typically the Euclidean distance, to identify the closest training points to a new query point. In KNN regression, the predicted value for a given query point is the average of the target values of its *k*-nearest neighbors. The hyperparameter *k* determines how many neighbors are considered in the prediction. Because KNN does not build a model, the computational cost of training is minimal, but predictions can be slower, especially with large data sets.

The algorithm for KNN can be explained as follows: for a given test point *x*, KNN calculates its distance to all training points using a predefined distance metric. The most common distance metric used is the Euclidean distance. Once the distances between the test point *x* and all training points are computed, the algorithm identifies the *k-*nearest neighbors by selecting the *k* points with the smallest distances. Let *N*_*k*_(*x*) be the set of *k*-nearest neighbors of *x*. Then, in KNN regression, the predicted value  for a new point *x* is computed as the average of the target values of its *k*-nearest neighbors:
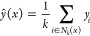
12

Artificial neural networks (ANNs) are a class of machine learning algorithms inspired by the structure and functioning of biological neural systems.^53^ ANNs learn complex patterns from data through interconnected layers of nodes or “neurons.″ These algorithms are widely used in tasks such as classification, regression, and generative modeling, especially when dealing with high-dimensional or unstructured data. ANNs consist of an input layer, one or more hidden layers, and an output layer. The hidden layers contain neurons that apply linear transformations followed by activation functions to the input data, allowing the network to model nonlinear relationships. The training process involves iteratively updating the network parameters (weights and biases) using optimization algorithms, e.g., stochastic gradient descent (SGD), to minimize a loss function, such as mean squared error (MSE) or cross-entropy loss. Nowadays, neural networks are the backbone of deep learning and a cornerstone of many state-of-the-art AI systems.^54^

The algorithm for training a neural network can be summarized as follows: given a data set of input–output pairs (*X,Y*), the network predicts outputs  by applying forward propagation through its layers. The error between the predicted and actual outputs is quantified using a loss function, and the gradients of this loss with respect to the network parameters are computed using backpropagation. Finally, the parameters are updated in the direction of the negative gradient by using an optimization algorithm. This process is repeated iteratively, until the model converges to an optimal set of parameters. The mathematical formulation for a single neuron in a neural network is where *x* is the input, *W* is the weight vector, *b* is the bias, *z* is the linear combination, and *f*(*z*) is the activation function. The activation function introduces nonlinearity, enabling the network to model complex patterns.
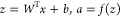
13

### 2.3 Isolation Forest for Anomalies Detection

Anomaly detection is a critical task across various domains, such as industrial monitoring, fraud detection, cybersecurity, and medical diagnostics. The objective is to identify data points that deviate significantly from expected patterns, which may indicate rare but important events such as system malfunctions, fraudulent activities, or network intrusions. This task is challenging due to the complexity and variability of real-world data, where normal behavior can fluctuate significantly and anomalies may be subtle or occur in high-dimensional spaces.

Isolation forest (IF), introduced by Liu et al.,^55^ offers a novel approach to anomaly detection by focusing on the concept of isolation rather than traditional distance or density-based measures. The core principle of IF is that anomalies are “few and different”, making them easier to isolate from the rest of the data. Instead of evaluating a point’s relative position within the data set, IF isolates each data point by recursively partitioning the data set through random splits. IF is an efficient algorithm for anomaly detection, focusing on isolating data points by recursively splitting the data set. Anomalies stand out because they differ significantly from normal data points, and as a result, they are isolated more quickly during the partitioning process.

The IF method offers several key advantages: (i) scalability: the algorithm scales linearly with the data set size, making it ideal for large-scale applications; (ii) no assumptions about data distribution: unlike methods that rely on specific data distribution assumptions (e.g., Gaussian), IF is distribution-agnostic, enhancing its robustness across various domains; (iii) handling high-dimensional data: IF performs effectively on high-dimensional data sets, avoiding the “curse of dimensionality” that hampers many traditional approaches.

The path length *h*(*x*) represents the number of edges traversed in an isolation tree before a point *x* is isolated. Anomalous points, which are more distinct, tend to have shorter path lengths. The anomaly score is based on this path length but normalized to fall within the range [−1, 1] where −1 indicates anomalies and 1 represents normal data points.

Given a point *x*, its anomaly score *s*(*x*) is calculated as
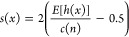
14

Where *E*[*h*(*x*)] is the average path length of point *x* across the isolation trees and *c*(*n*) is the normalization factor, representing the average path length for a normal point in a data set of size *n*. The IF algorithm operates as follows: data set *X* of size *n* and a number of trees *t* are subsampled with size ψ, forming a subsample *X*_ψ_. Then the subsample is recursively partitioned with a selected feature *f* and a split value *p* within the range of the feature, until each data point is isolated or the tree reaches a maximum depth *L*. For each point *x*, traverse the isolation tree to compute its path length *h*(*x*). Finally, calculate the anomaly score *s*(*x*), if it is positive, it indicates a normal point; on the contrary, if negative, it represents an anomaly.

## 3 Results and Discussion

The sensor data sets were collected from open-loop experiments, as previously described and consistent with our earlier study.^50^ To reduce noise and ensure comparability, the data sets were normalized, and a 1200-point moving average filter was applied to the data-driven fault prediction outputs, and residual signals were filtered using fast Fourier transform (FFT). Data-driven training and analysis were conducted on a Lenovo ThinkPad P53 (Intel 9850H CPU, Nvidia Quadro RTX 5000 mobile GPU, 128 GB RAM) using Python (Scikit-learn, TensorFlow, and PyTorch) and MATLAB on Slackware Linux. Detailed parameters for RF, KNN, and IF are provided in Table S2 and ANN and RNN are provided in Table S3.

### 3.1 Data-Driven Fault Diagnosis

The residual generators have demonstrated their ability to detect, isolate, and estimate faults in the CSTR process. Following the experimental runs, the collected data prompted the question of whether data-driven methods could also be applied for fault diagnosis. A total of 19 data sets with sensor readings and known fault sizes were used, with one serving as the test set and the remaining 18 as the training set. We applied two methods: random forest (RF) and *k*-nearest neighbors (KNN). RF is a model-based statistical approach, while KNN is nonparametric, providing a representative comparison for testing data-driven methods.

Since we already know the exact time and magnitude of the faults from the experimental data, training the data-driven models does not require knowledge of the system equations(eqs 1–5) or the model-based residual generators (eqs 6–9). Instead, the sensor readings are used to train the models solely on the basis of the fault occurrence times and fault sizes, making this a “model-less” approach to fault diagnosis. The detection thresholds were determined using a Bayesian changepoint detection mechanism^56^ with one data set and tested across all data sets, ensuring effective and accurate fault detection. Additionally, the data-driven regressors accurately converged to the actual fault size, demonstrating a strong performance in fault estimation.

A comprehensive analysis was conducted as follows:

a. Only one fault is happening, 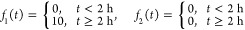
With only one fault introduced into the system—fault 1, caused by a spike in coolant inlet temperature—the expected outcome is accurate detection and estimation of the fault size, while predictions for fault 2 remain low, as no fault associated with 3-picoline concentration is present.

**Figure 3:**
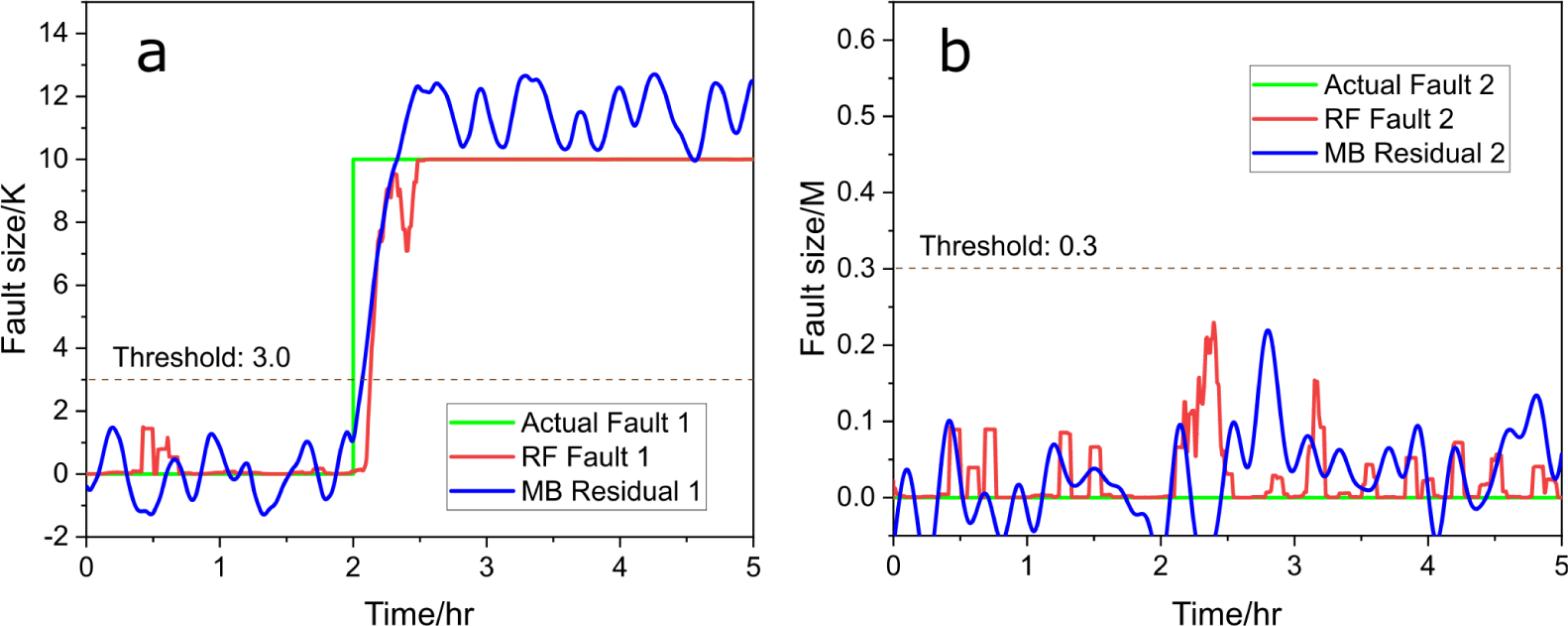
Predicted fault size (red) vs actual fault size (green), (a) for fault 1 and (b) for fault 2, with RF and compared to model-based (MB) residual generators (blue), under scenario a.

As shown in Figure 3a, fault 1 is introduced at the 2 h mark, at which point the RF regressor (red line, eq 11) promptly detects the fault (represented by the green line) and responds within 5 min, converging to the correct fault size of 10 within 30 min. A threshold of 3 could be applied for fault detection, when the signals of the residual generator or RF regressor exceed this threshold, a fault is then alarmed. We also evaluated the performance of the moving average filter, with a 1200-point window proving to be an effective choice, with *R*^2^ = 0.96, and the results are provided in Table S3. The performance of RF is comparable to that of a model-based residual generator (blue line, “MB” for model-based) with a slight delay, demonstrating the efficiency of RF in fault detection and size estimation. In Figure 3b, the signal for fault 2 remains low throughout the test, confirming that no fault related to the decrease in 3-picoline concentration is present, of which the residual generator (blue line) provides a similar result. Similarly, a threshold could be set at 0.3, and both signals remain below this threshold, indicating that there is no fault happening. This accurate isolation further validates the robustness of the RF model for single-fault scenarios. These results highlight the ability of data-driven methods to both detect and isolate faults with precision comparable to model-based residual generators, even in complex system dynamics.

**Figure 4:**
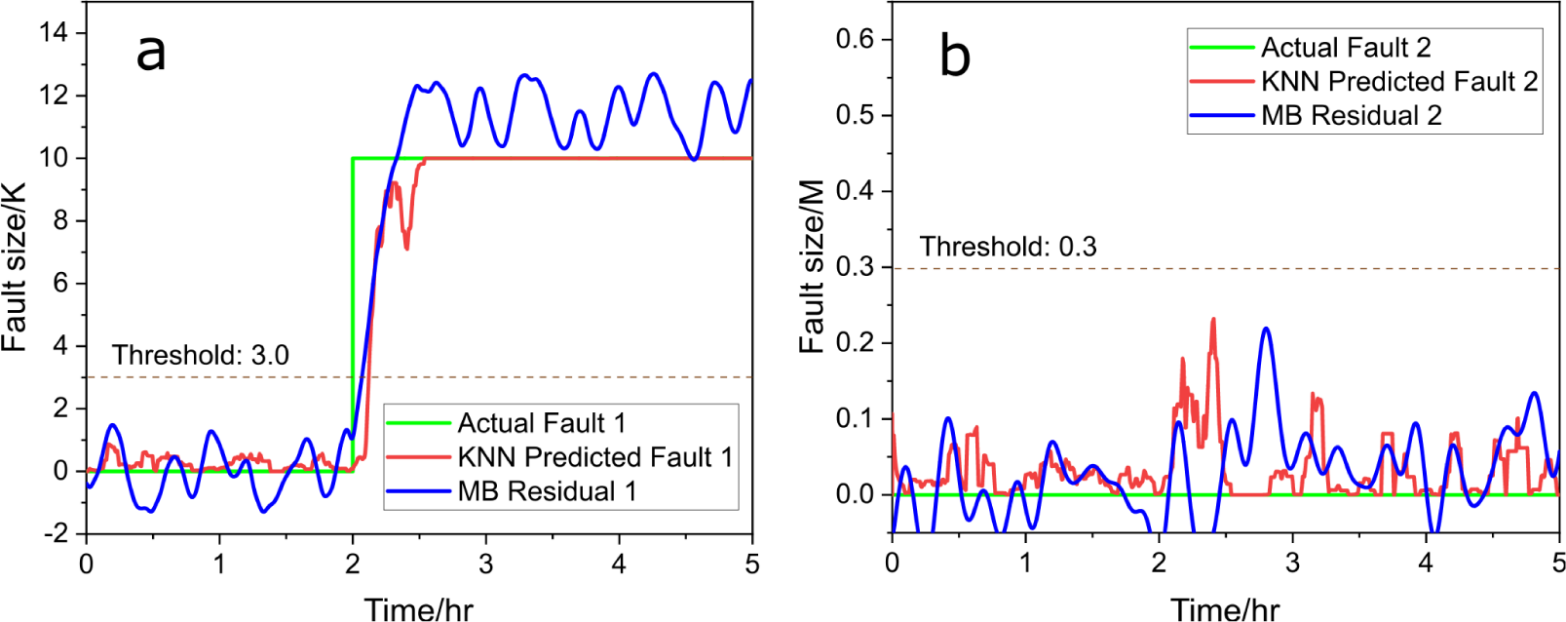
Predicted fault size (red) vs actual fault size (green), (a) for fault 1 and (b) for fault 2, with KNN and compared to model-based (MB) residual generators (blue), under scenario a.

The same procedure was applied using the *k*-nearest–neighborhood (KNN) regressor, and the results are shown in Figure 4. In Figure 4a, the KNN model (red line, eq 12) swiftly responds to the introduced fault 1, similar to the RF results, detecting the fault promptly and converging toward the correct fault size. In Figure 4b, the signal for fault 2 remains consistently low throughout, indicating successful fault isolation and confirming that no fault is associated with the decrease in the 3-picoline concentration. These results demonstrate that the KNN regressor, like the RF model, achieves both fault isolation and size estimation effectively, reinforcing the viability of data-driven approaches in fault diagnosis tasks. In both methods, there is a slight delay of data-driven methods compared with model-based residual generators, showing a possible quicker response in residual signals.

b. Only one fault is happening, 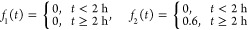


In the reversed scenario, where the only fault occurring in the system is fault 2—a decrease in the 3-picoline feed inlet concentration—the expectation is that the model will detect and estimate the size of fault 2, while the prediction for fault 1 remains low, as no coolant inlet temperature spike is present.

**Figure 5:**
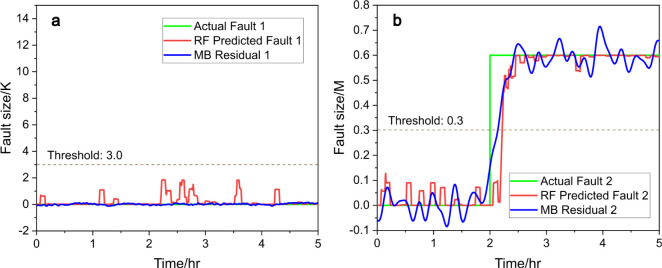
Predicted fault size (red) vs actual fault size (green), (a) for fault 1 and (b) for fault 2, with RF and compared to model-based (MB) residual generators (blue), under scenario b.

**Figure 6:**
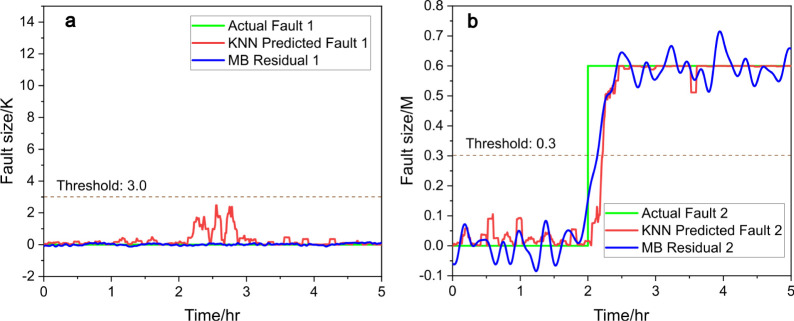
Predicted fault size (red) vs actual fault size (green), (a) for fault 1 and (b) for fault 2, with KNN and compared to model-based (MB) residual generators (blue), under scenario b.

In Figure 5a, the fault 1 prediction remains consistently lower than 3, indicating no fault related to the coolant inlet temperature, as expected. In Figure 5b, the model responds to fault 2 swiftly and converges to the correct fault size within 30 min, showing promising results in fault detection and estimation. Similarly, when the KNN regressor is tested in this scenario: in Figure 6a, the predicted value for fault 1 stays low, though slightly higher than that of the RF model, still confirming no fault. In Figure 6b, the KNN model promptly detects fault 2, accurately estimating its size with a short response time.

**Figure 7:**
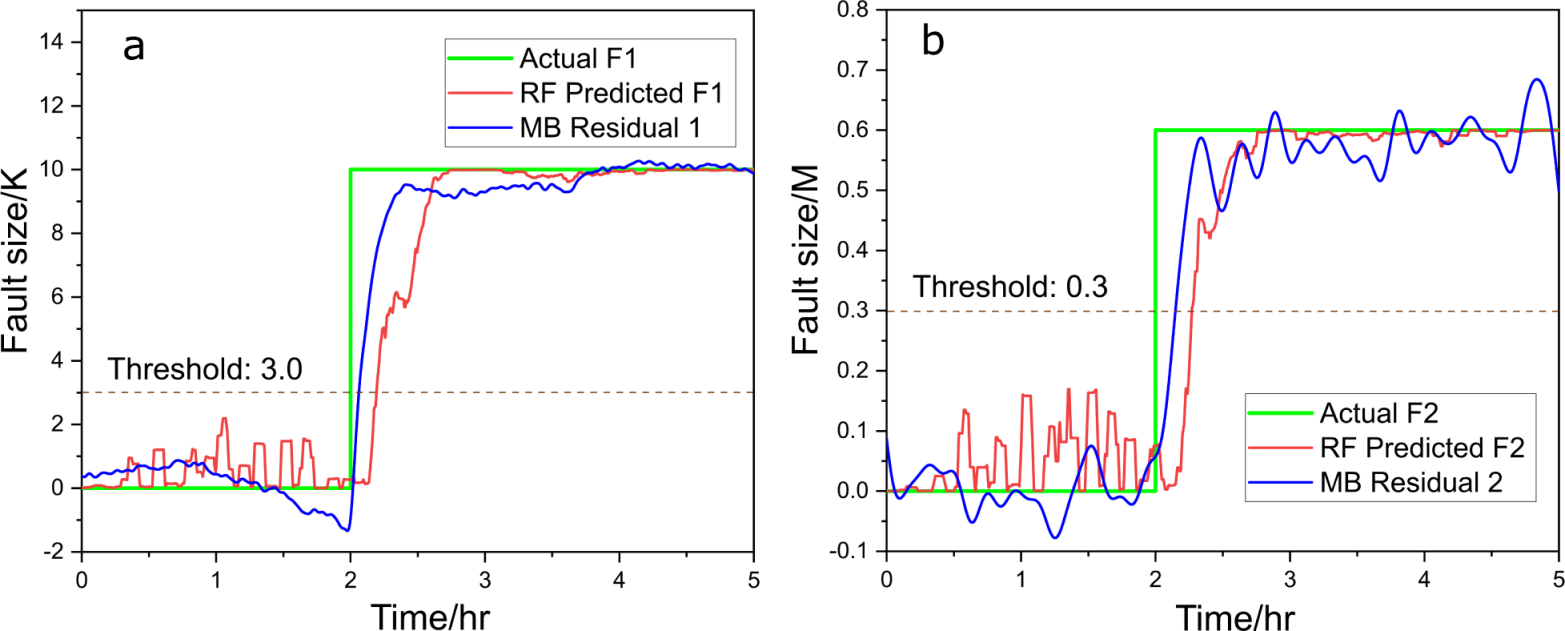
Predicted fault size (red) vs actual fault size (green), (a) for fault 1 and (b) for fault 2, with RF and compared to model-based (MB) residual generators (blue), under scenario c.

**Figure 8:**
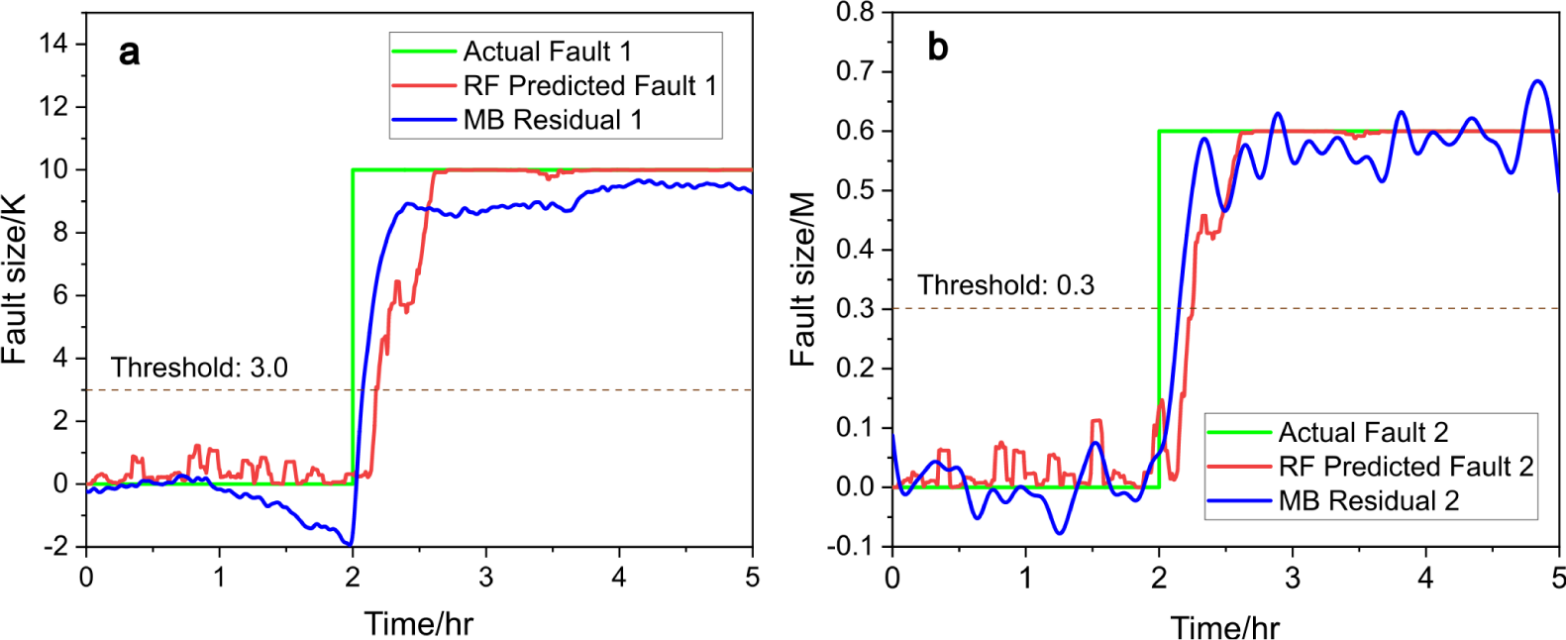
Predicted fault size (red) vs actual fault size (green), (a) for fault 1 and (b) for fault 2, with KNN and compared to model-based (MB) residual generators (blue), under scenario c.

Despite minor discrepancies observed in both fault scenarios, the models consistently detect faults when they occur, although the precision of the magnitude estimation varies. Crucially, the timing of fault detection is accurate in both cases, with the models successfully identifying both the onset and the resolution of faults. This is especially clear in the case of fault 2, where the predicted and actual fault sizes nearly converge during the period of sustained fault, demonstrating the models’ effectiveness in accurately tracking fault behavior over time. For both RF and KNN, a slight delay is also present compared with residual signals.

c. Two faults are happening, 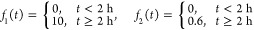


Figures 7 and 8 present the comparison between the actual and predicted fault sizes for fault 1 and fault 2, using two different machine learning models: RF and KNN, respectively. Each figure consists of two subplots: Figures 7a and 8a represent fault 1, and Figures 7b and 8b represent fault 2. In both cases, the predictions are compared against the ground of fault sizes, along with model-based residual signals.

The random forest model shows a very close alignment between the predicted fault size (red line) and the actual fault size (green line). The model captures the onset of the fault around 2 h, and the prediction remains accurate throughout the fault duration. As for the residual signal, there is a small deviation, less than 1.0 K, especially during the sustained fault event (fault size ∼10), which is acceptable. The sharp rise and stable fault size during this period suggest that random forest handles significant faults efficiently and with high precision. Prior to the major fault event, the RF model exhibits some small fluctuations in the predicted fault size that occur in the time range between 0 and 1.5 h. These small deviations do not affect the overall value as they are below the detection threshold.

The KNN also shows strong predictive performance with the predicted fault size (red line) following the actual fault size (green line) very closely during the main fault period. The rise of the fault at approximately 2 h and the sustained fault are captured well.

For fault 2, both models exhibit similar performance, but the random forest model shows more variability and fluctuation in its predictions. This might indicate that random forest is more prone to overfitting to noise in cases where the fault dynamics are more complex, but all fluctuations are well below detection thresholds. As for the model-based residual signal (MB, the blue line), the noise level is much higher than data-driven methods; however, it has a better prompt response to the fault happening.

Neural networks have gained popularity in recent years due to their ability to model complex, nonlinear systems effectively. Building on the fault diagnosis conducted using KNN and RF, we also applied artificial neural networks (ANN) and recurrent neural networks (RNN) to explore their effectiveness. These methods were chosen to leverage their capacity for capturing intricate patterns and, in the case of RNN, for addressing temporal dependencies within the data.

**Figure 9:**
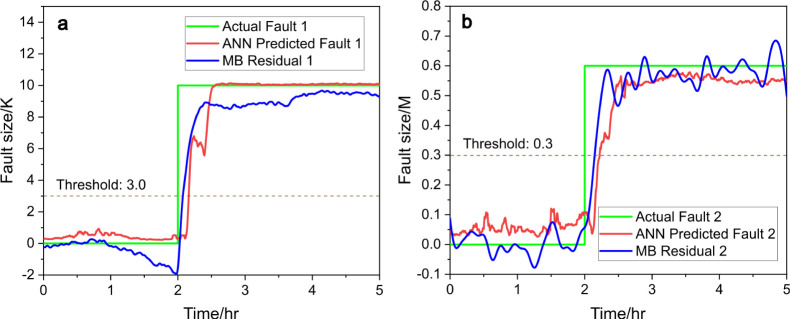
Predicted fault size (red) vs actual fault size (green), (a) for fault 1 and (b) for fault 2, with ANN and compared to model-based (MB) residual generators (blue), under scenario c.

**Figure 10:**
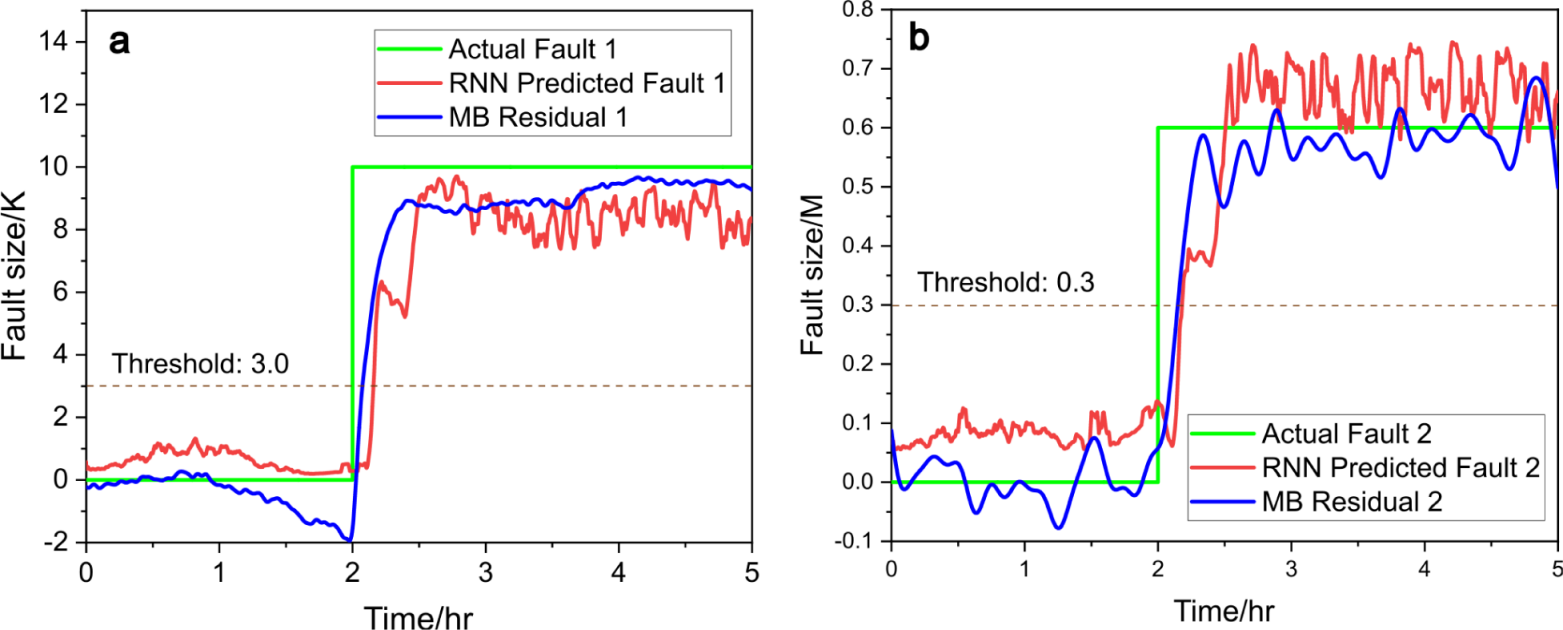
Predicted fault size (red) vs actual fault size (green), (a) for fault 1 and (b) for fault 2, with RNN and compared to model-based (MB) residual generators (blue), under scenario c.

The fault diagnosis achieved using an ANN and an RNN is illustrated in Figures 9 and 10. In Figures 9a and 10a, the ANN and RNN successfully identify fault 1 by comparing the actual fault (green line) with the predicted fault (red line). The threshold of 3.0 (dotted line) is used to detect fault occurrences. Similarly, in Figures 9b and 10b, fault 2 is diagnosed with a threshold of 0.3, where the ANN and RNN both capture the fault dynamics through the alignment of the actual fault (green line) and predicted fault (red line). This demonstrates the effectiveness of neural networks in detecting and predicting fault conditions. Figure 10 plotted using RNN appears to exhibit higher noise levels in the predicted faults compared with Figure 9. This increased noise could be attributed to RNN’s sensitivity to temporal dependencies, which may amplify variations in the data.

While the neural network demonstrates strong performance in fault diagnosis, the computational time required is much higher compared to RF and KNN. The average time consumption for a single cycle of training and testing using KNN, RF, ANN, and RNN is detailed in Table S5. Additionally, Table S6 compares the performance of these methods in estimating fault sizes against the actual fault values. We will present results only for KNN and RNN, as they demonstrate satisfactory performance.

d. Two faults happening at different times, 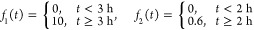


It is crucial to verify fault isolation with experimental data that fault 1 and fault 2 are happening at two different times.

**Figure 11:**
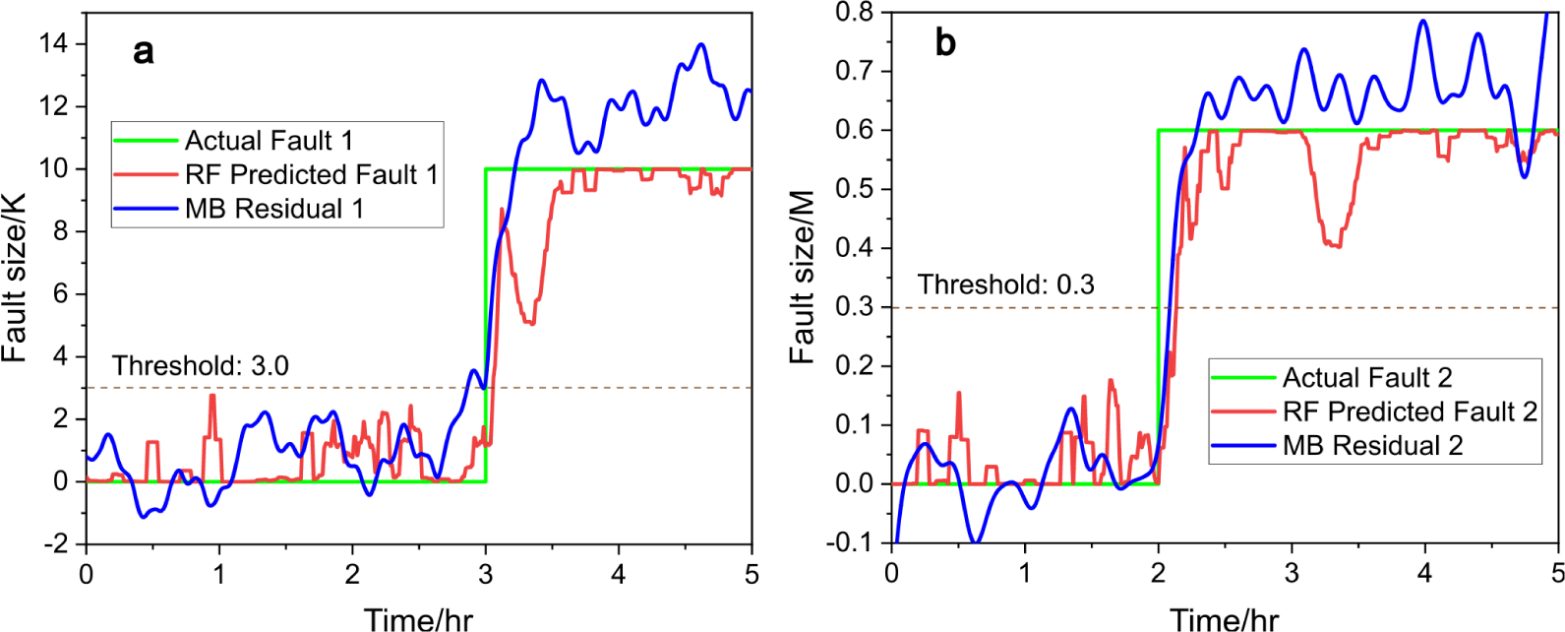
Predicted fault size (red) vs actual fault size (green), (a) for fault 1 and (b) for fault 2, with RF and compared to model-based (MB) residual generators (blue), under scenario d.

**Figure 12:**
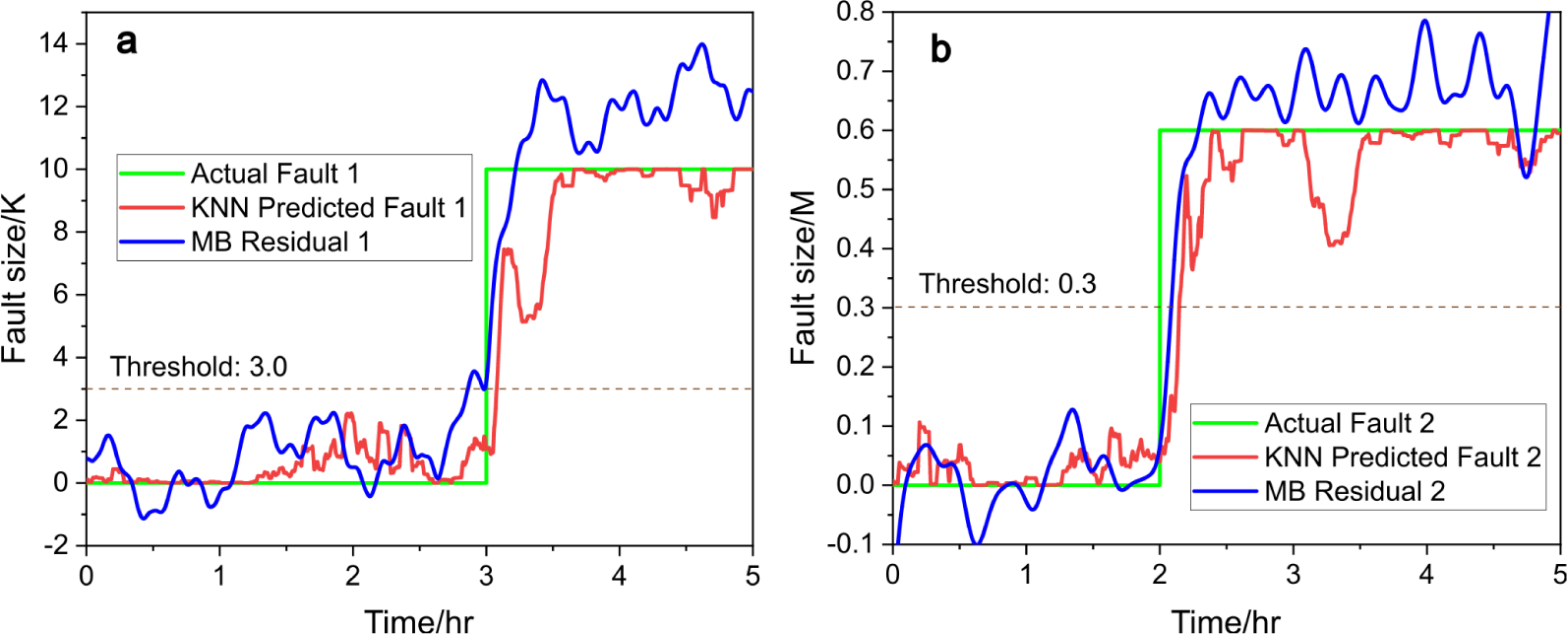
Predicted fault size (red) vs actual fault size (green), (a) for fault 1 and (b) for fault 2, with KNN and compared to model-based (MB) residual generators (blue), under scenario d.

The RF model accurately predicts the onset and magnitude of fault 1 in Figure 11a. The predicted fault size (red line) closely follows the actual fault size (green line), particularly during the critical period after 3 h, where the fault size rises sharply and stabilizes around a fault size of 10. The model consistently captures the duration and magnitude, demonstrating a high prediction precision during the main fault event. The KNN model similarly demonstrates strong predictive performance for fault 1 in Figure 12a, with the predicted fault size (red line) following the actual fault size (green line) closely. The model effectively captures the sharp rise in the fault size around 3 h, maintaining accuracy throughout the sustained fault period. As for the model-based residual signal (MB, the blue line), we observe an overshoot, giving a 1.2 K overestimation of fault size, which is within the experimental tolerance.

For fault 2, in Figure 11b, the predicted random forest model fault size (red line) captures the general trend of the fault, which begins around 2 h and persists until the end of the test. After the fault reaches its maximum (fault size 0.6), the random forest model stabilizes and follows the actual fault size (green line) more closely. In Figure 12b, the KNN model offers slightly smoother predictions for fault 2. While the predicted fault size (red line) does fluctuate in the early fault period (around 3 h), the overall estimation is sound.

**Figure 13:**
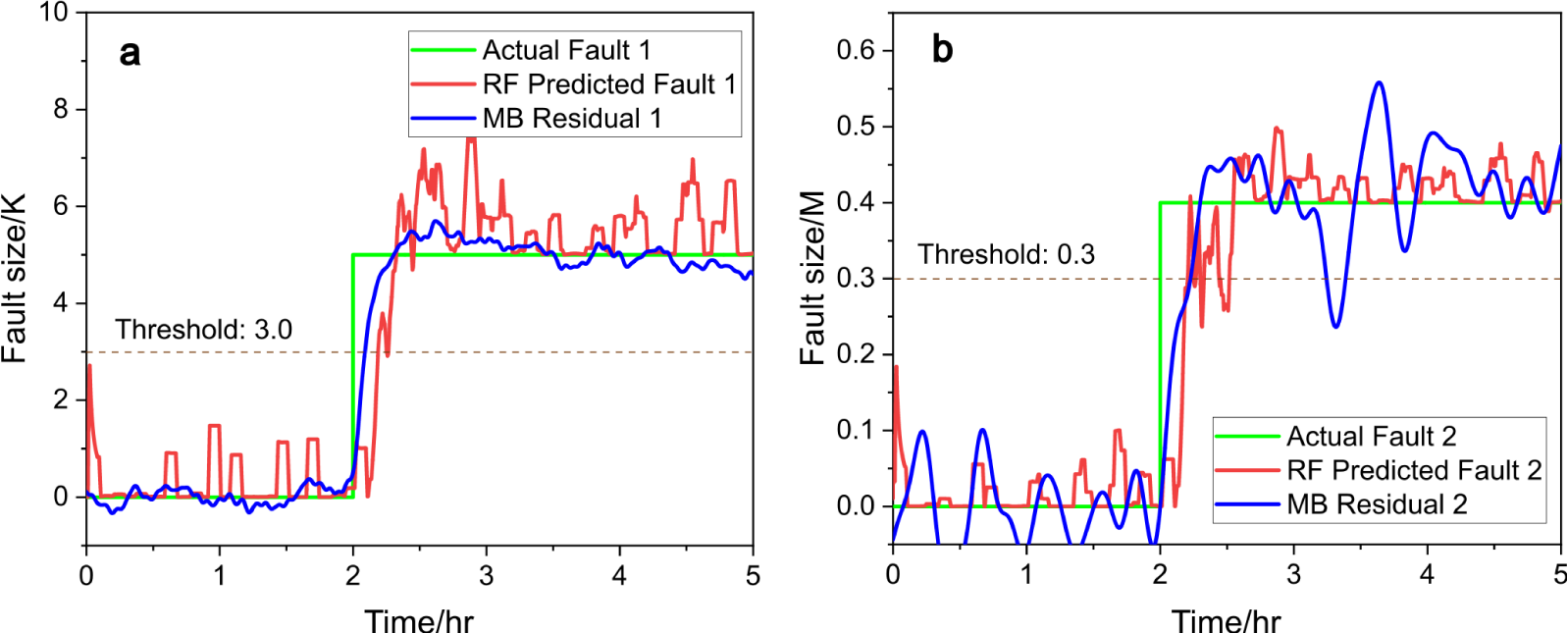
Predicted fault size (red) vs actual fault size (green), (a) for fault 1 and (b) for fault 2, with RF and compared to model-based (MB) residual generators (blue), under scenario e.

**Figure 14:**
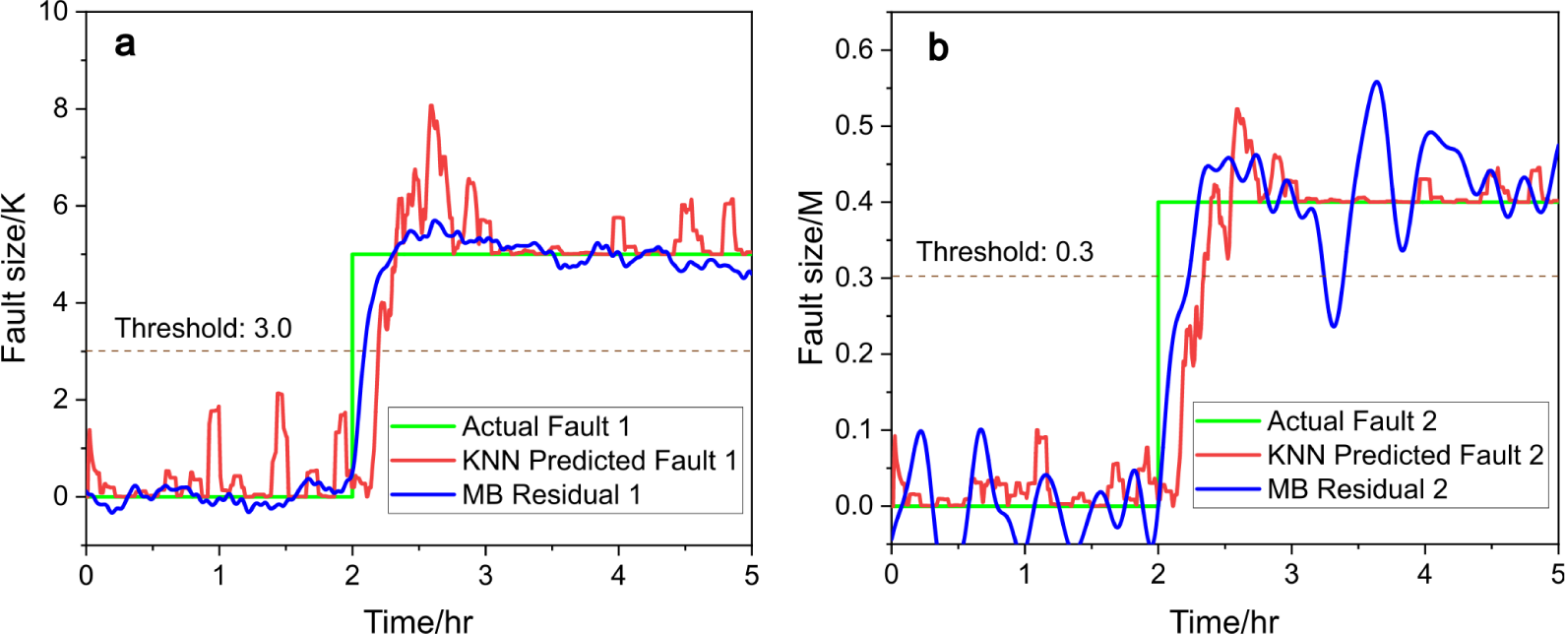
Predicted fault size (red) vs actual fault size (green), (a) for fault 1 and (b) for fault 2, with KNN and compared to model-based (MB) residual generators (blue) under scenario e.

These results exhibit successful fault isolation; the noise level is not too high to invalidate fault detection, and especially the estimation for both fault sizes is remarkably good. The slower response compared with residual generators is not unforeseeable, as model-based methods have the edge in the system dynamics.

e. Two smaller faults are happening, 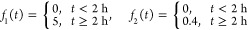


Figures 13 and 14 demonstrate the performance of fault prediction models RF and KNN for a smaller fault scenario, respectively. Each figure consists of two subplots: a represents fault 1 and b represents fault 2, where the faults are smaller in magnitude compared to previous scenarios.

In this smaller fault scenario, the random forest model shows relatively good performance in Figure 13a. The predicted fault size (red line) aligns with the actual fault size (green line), particularly after the fault onset around 2 h. However, compared to the larger fault scenario, the random forest model introduces more variability in its predictions. The predicted fault size fluctuates around the actual fault size, especially after 2.5 h, where the model tends to overestimate the fault magnitude. The KNN model provides more stable predictions for fault 1 under the smaller fault scenario in Figure 14a after 3 h. The predicted fault size (red line) follows the actual fault size (green line) closely, with fewer deviations compared with the random forest model. There is a minor overestimation of the fault size around 2.5 h, but overall, the model tracks the actual fault size more consistently. The residual signal shows superb performance with both accuracy and responsiveness.

For fault 2, the random forest model (Figure 13b) and the KNN model (Figure 14b) exhibit a slight overestimate at the beginning of the fault occurrence , and the KNN model shows a larger overshoot at around 2.5 h. Overall, the KNN model’s ability to provide smoother predictions with fewer oscillations makes it better suited for capturing the smaller faults in fault 2, as it appears less sensitive to noise or minor deviations in the data especially after 3 h.

Estimating smaller faults is generally more challenging due to the lower signal-to-noise ratio, a difficulty also noted with model-based observers, as discussed in our previous study.^50^ The results demonstrate that data-driven methods (RF and KNN) are capable of diagnosing these minor faults effectively.

In conclusion, both models are effective at detecting smaller faults. Model performance: both random forest (RF) and *k*-nearest neighbors (KNN) demonstrated strong performance in detecting and isolating faults as well as estimating their sizes. The circular iteration ensured robustness and consistency across the data set. However, in all the cases, model-based residual signals show better responsiveness and faster fault detection. Accuracy: the average accuracy across all tests was high, with minimal variance between data sets. This indicates the effectiveness of both RF and KNN across different operational conditions. Fault Isolation: both methods successfully isolated the faults in the system, identifying the affected components without false positives or significant misclassifications. Fault size estimation: the size estimation of faults was within acceptable error margins, showing that data-driven methods, when properly trained, can provide reliable fault magnitude estimates without relying on model-based equations.

### 3.2 Isolation Forest for Anomalies Detection

A major limitation of the model-based approach is that not all processes can be easily modeled, and even with a validated model, operational parameter changes may still occur. Similarly, data-driven methods cannot reliably diagnose faults without prior knowledge of faulty data sets or at least similar conditions for training. For example, during experimentation, it was observed that after reassembling the equipment the previously effective model-based residual generators showed reduced performance, while data-driven methods produced false detections. This setup considered a scenario in which only a single fault was present in the process, as described below. 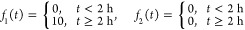.

**Figure 15:**
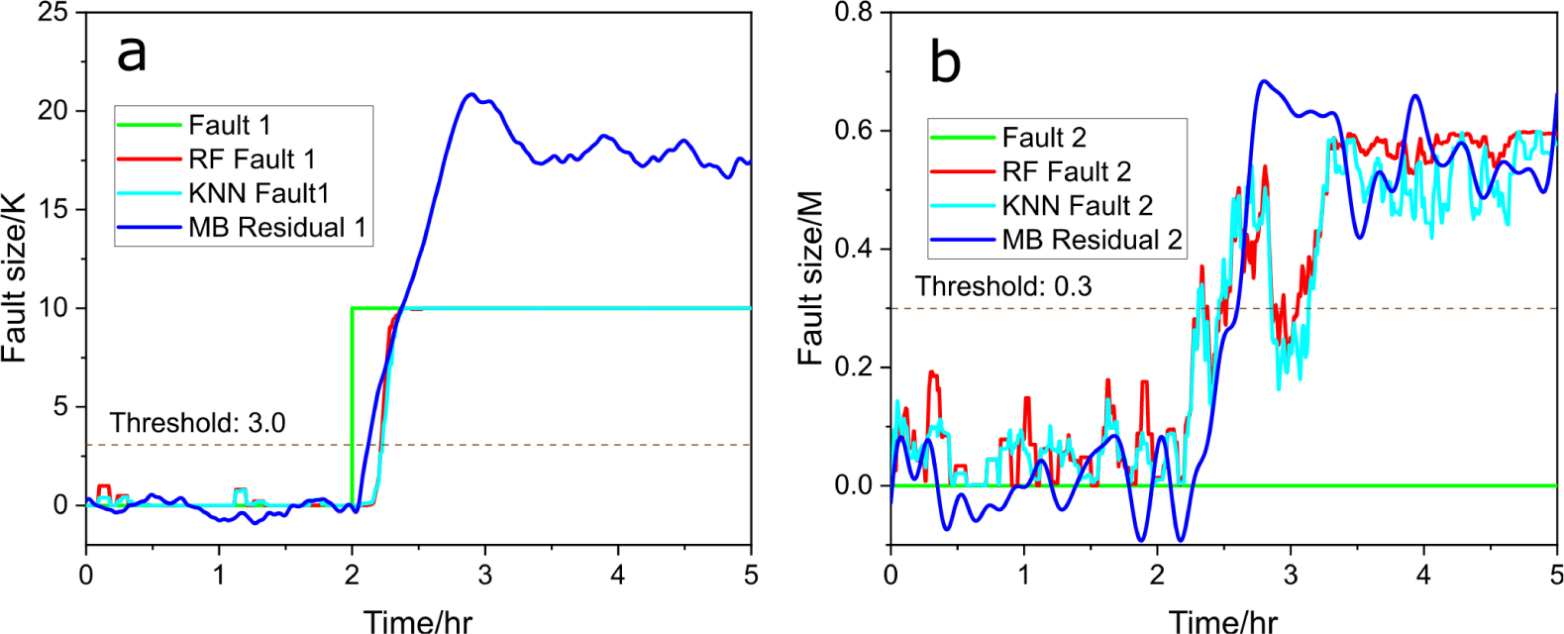
Predicted fault size vs actual fault size with residual generators, RF (red) and KNN (cyan), (a) for fault 1 and (b) for fault 2, and compared to model-based residual generators (blue).

The model-based residual signals are shown as blue lines. For fault 1 in Figure 15a, the model-based residual signal spikes above 20 before stabilizing around 17, which is over 60% higher than the actual fault size, indicating a clear overestimation. In Figure 15b, despite no fault being present for fault 2, the residual signal incorrectly estimates around 0.5, as a false positive. For the data-driven methods, RF (red lines) and KNN (cyan lines), the performance for fault 1 in Figure 15a is notably strong, accurately detecting and estimating the fault size at 10. However, both methods also produced false positives for fault 2 in Figure 15b. All methods failed under the system parameter change, which is expected. Data-driven methods struggled due to the lack of similar scenarios in the training sets, and the model-based observer failed because the parameter change caused a mismatch in the system model, invalidating the residual signals.

This was traced back to a change in the heat transfer coefficient with additional experiments. It is crucial to detect changes in the heat transfer coefficient (*U*) before any faults are introduced into the system. Early detection allows for timely adjustments to the model parameters, preventing potential losses due to detection delays when actual faults occur. Therefore, the goal is to detect shifts in *U* during the nonfault steady-state operation, enabling proactive model adjustments before faults impact system performance.

In data-driven methods, detecting changes in the heat transfer coefficient (*U*) is equivalent to identifying anomalous data sets compared to a set of nominal runs. Therefore, it is natural to apply an anomaly detection algorithm, with Isolation forest (IF) being an ideal choice due to its simplicity and low computational resource requirements. The isolation forest (IF) parameter settings include 200 estimators, a contamination level of 0.2, and a maximum sample size of 256. Further details on all parameter settings are provided in Table S2. The sensor data sets, collected before any faults were introduced into the system and with a known *U =* 18 W/(m^2^·K) (calculated using a heat transfer area of 0.08 m^2^), are used to train the IF model. After training, several data sets with potentially altered *U* values are tested against the model, and anomaly scores are calculated according to eq 14.

**Figure 16:**
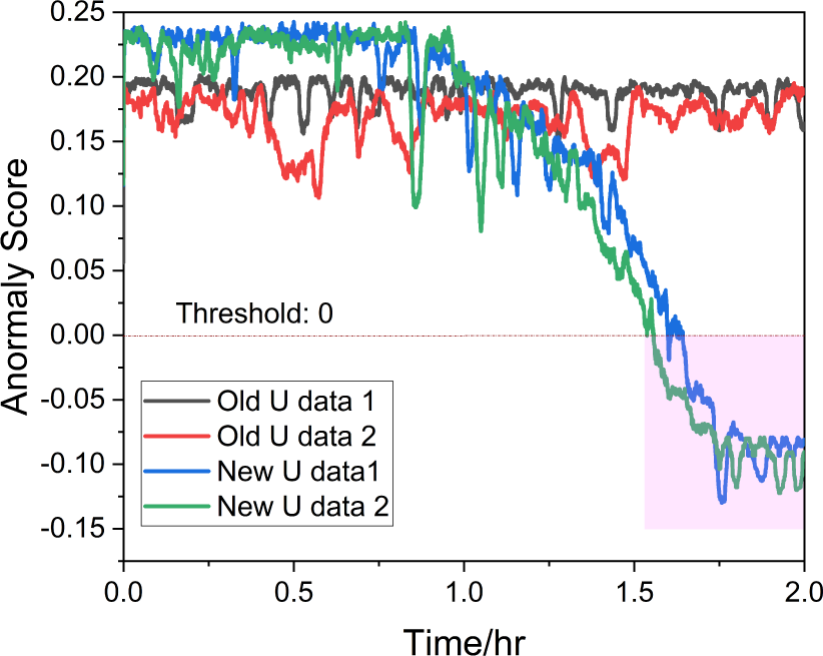
Isolation forest anomaly score for process *U* change.

The results are summarized in Figure 16, where four data sets were randomly selected for testing: two with old *U =* 18 W/(m^2^·K) and two with new *U*. In the figure, the two data sets with *U =* 18 show consistently positive anomaly scores, indicating that the sensor readings align with the training set. In contrast, the two data sets with altered *U* values show a gradual detection of anomalies by the IF model, with anomaly scores dropping sharply into the negative range and stabilizing around −0.1, as marked by the pink area in Figure 16. This successful anomaly detection confirms a change in *U* within these two data sets.

Once an anomaly is detected, the next step is to adjust the model parameter (*U*) to ensure that the residual generators continue to function optimally for fault diagnosis. There are numerous regressors available in the data-driven toolbox for estimation tasks. However, a key drawback of data-driven methods is that models must be trained with data sets corresponding to various known *U* values. In practice, the *U* values may not always be known *a priori*, which can result in unreliable output estimates. To address this, we refer back to the system model described in eqs 1–5.

Assuming the system is in a steady state and no faults are present, we can derive an equation from eqs 1 and 3, resulting in eq 15. Additionally, by considering eq 4 alone, we can establish eq 16. Both of these equations provide estimates of the *U* value, offering a more reliable approach than purely data-driven methods.
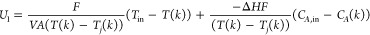
15
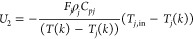
16

**Figure 17:**
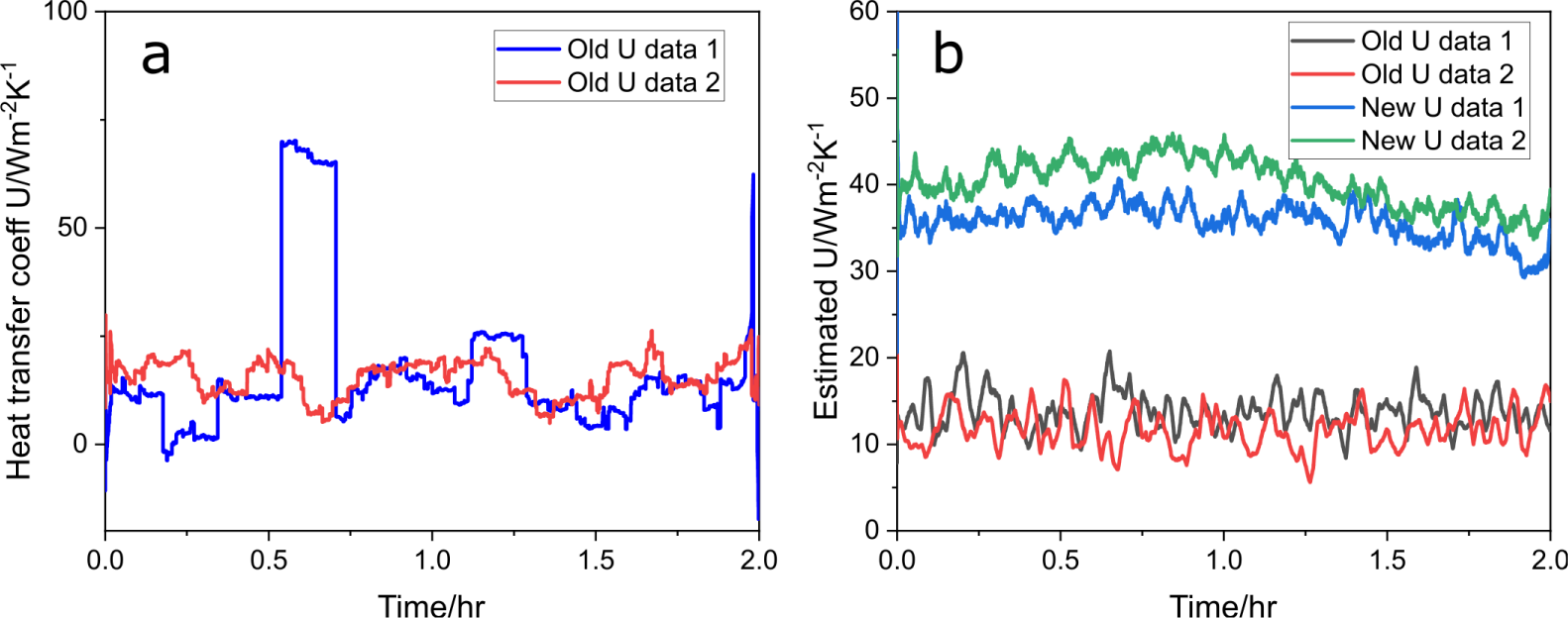
Direct calculation of *U* values for two nominal runs (a) and optimization estimates (b).

Direct calculation using eq 16, as shown in Figure 17a, incorporates a moving average filter with a window size of 6000 points. However, the results are suboptimal due to the high noise levels. Not all data sets can be reliably calculated using eqs 15 and 16 because the divisor involves temperature measurements, which are subject to sensor noise. As noted in eq 5, floating-point division can lead to significant errors when the divisor is small.

The mean values of the calculated lines in Figure 17a are 17.6 and 19.07, which are close to the actual value of 18 W/(m^2^·K). However, the high noise level makes direct calculation impractical for all data sets. This issue arises because the reactor jacket’s thickness is only around 0.2 mm, resulting in a large heat transfer coefficient. Consequently, the temperature difference between *T* – *T*_*j*_ is small and is exacerbated even more, as the measurement noises (eq 5) of temperature have a variance over 0.6, which can lead to significant errors in the calculation due to the small magnitude of the temperature difference.

A more effective approach is to frame the problem as an optimization task. By rearranging eqs 15 and 16, we derive the residuals as follows:
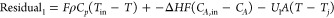
17
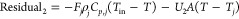
18

The objective function to be minimized is the sum of the squared residuals:
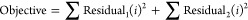
19under the condition with the lower bound [*U*_1_*U*_2_] = [0 0].

The optimization problem is solved using the global trust region reflective algorithm, and the results are plotted in Figure 17b. The calculated curves exhibit much smoother behavior. The nominal runs, old *U* data 1 (black line) and old *U* data 2 (red line), fluctuate between 15 and 20, indicating stable but slightly lower *U* estimates over the actual value of 18. The overall trend for these nominal conditions remains relatively constant.

In contrast, the changed conditions, new *U* data 1 (green line) and new *U* data 2 (blue line), show fluctuations around 40 W/(m^2^·K), representing significantly higher *U* values compared to the nominal runs. This stark difference in *U* values between the old *U* and new *U* conditions reflects a change in the heat transfer coefficient. The mean values of Figure 17b, calculated *U*, are summarized in Table 1.

**Table 1:** Calculated *U* Values from Optimization

Parameter	Old *U* data 1	Old *U* data 2	New *U* data 1	New *U* data 2
*U*/*W*/(m^2^K)	16.57	17.74	35.6	33.1

Using this method, following data triage, the experimental data revealed two distinct categories of *U* values: one centered around 18 W/(m^2^·K) (average 17.8, standard deviation 1.12) and another centered around 40 W/(m^2^·K) (average 38.9, standard deviation 3.1), demonstrating good stability. After isolation forest (IF) anomaly detection, if a data set is flagged as negative, the *U* value is recalculated to update the model-based residual generators for fault diagnosis.

**Figure 18:**
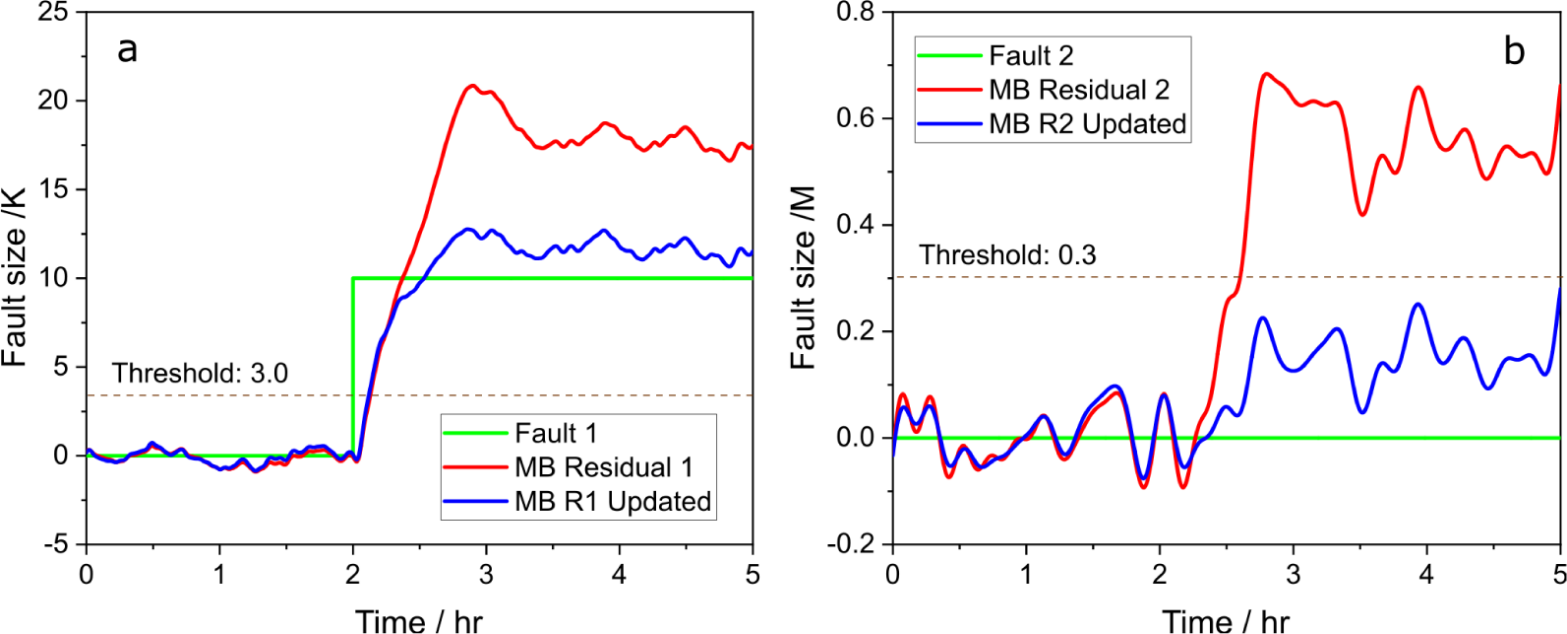
Model-based residual signal responses after parameter update (blue) and before update (red) compared with actual fault size (green).

The results are depicted in Figure 18, showing the fault diagnosis for both fault 1 and fault 2 using parameter-updated model-based residual generators. In Figure 18a on the left, the blue line represents the updated residual signal, which slightly overestimates the fault size after the 2 h mark, stabilizing around 11.5, within acceptable experimental tolerance. The red line shows the previous residual signal, as seen in Figure 15a, which deviates significantly from actual fault 1 (green line). In Figure 18b on the right, the results for fault 2 detection are shown, with no actual fault present (as indicated by the green line staying flat at zero). The updated residual signal (blue line), although showing some fluctuations, does not exceed the fault detection threshold. This updated signal performs much better than the previous residual signal in Figure 15b, indicated by the red line.

**Figure 19:**
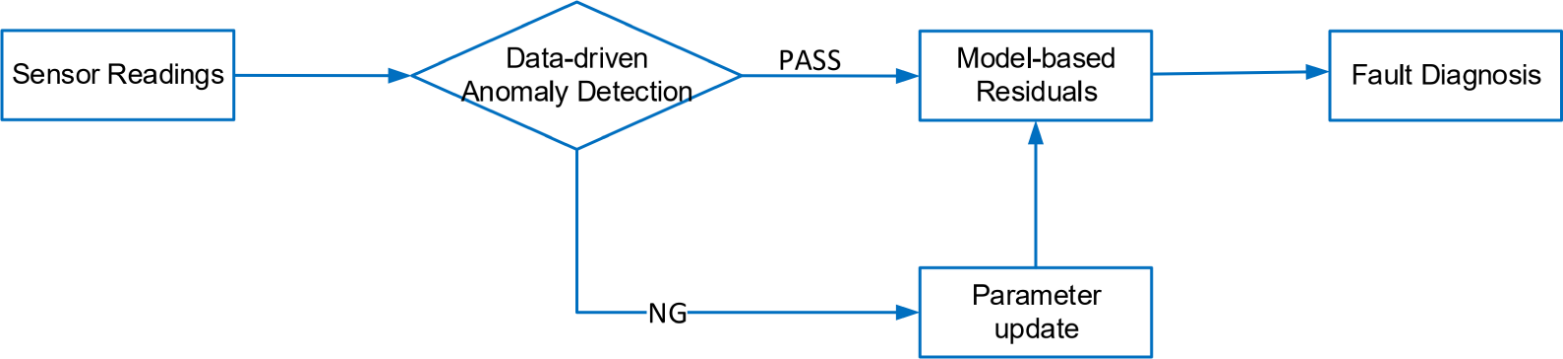
Flowchart of parameter update for model-based fault diagnosis.

The procedure is outlined in the flowchart presented in Figure 19. Initially, sensor readings are analyzed using the isolation forest (IF) algorithm for anomaly detection. If no anomalies are found, the system proceeds with the model-based residual generators using the default *U* value. However, if the anomaly scores drop below zero, indicating a potential issue, then an optimization algorithm is triggered to recalibrate the *U* value. This updated *U* value is then used to adjust the residual generators, allowing the model-based fault diagnosis to continue accurately under the new conditions.

To apply a data-driven method, a backup model-based residual generator must be used for fault diagnosis in the new parameter-changed scenario until sufficient data sets with the new parameter and fault conditions are collected. Once these data sets are validated against actual faults, they are incorporated into the data-driven RF and KNN training sets, labeled with the corresponding *U* value. This process updates the training sets, enabling data-driven methods to adapt to the new conditions.

**Figure 20:**
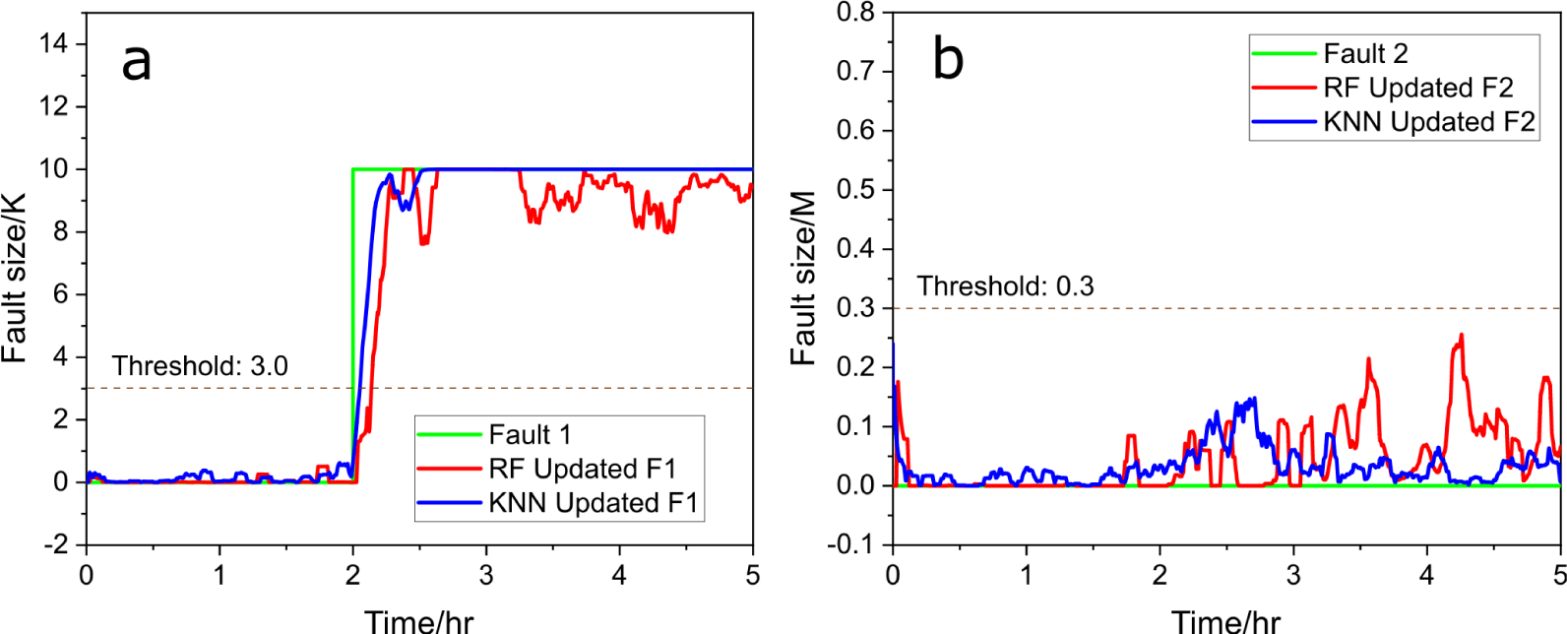
Predicted fault size vs actual fault size (green) with RF (red) and KNN (blue), (a) for fault 1 and (b) for fault 2 after training sets updated.

To evaluate the efficacy of this approach, seven data sets with *U =* 40 W/(m^2^·K) were used. Figure 20 presents the fault diagnosis results with RF and KNN. The same data set from Figure 13 is used as the testing set, and six new data sets marked with *U =* 40 were incorporated into the data-driven training set. This process allowed for data-driven approaches to train and gain new knowledge about the system, thus enhancing fault diagnosis.

The result is shown in Figure 20, presenting the fault diagnosis results for both fault 1 and fault 2 using training updated RF and KNN. In Figure 20a on the left, the red line shows the RF model’s prediction, which closely follows the actual fault size but fluctuates slightly after the initial detection, oscillating just below the true fault magnitude. The blue line, representing the KNN prediction, also aligns well with the actual fault, responding quickly and providing accurate estimates with fewer fluctuations compared with the RF prediction. In Figure 20b on the right, the results for fault 2 detection are displayed, where no actual fault is present (as indicated by the green line remaining flat at zero). Both the RF (red line) and KNN (blue line) predictions show minor fluctuations hovering slightly above zero. Nevertheless, these signals remain below the threshold of 0.3, indicating that there is no fault.

As more training scenarios are gathered, it has been demonstrated that data-driven methods can also effectively perform fault diagnosis, matching the reliability of model-based approaches. Overall, the data-driven approaches (RF and KNN) demonstrate strong performance for fault 1, accurately detecting and estimating the fault size. In the case of fault 2, despite some fluctuations, there are no false positives and reliable fault isolation.

**Figure 21:**
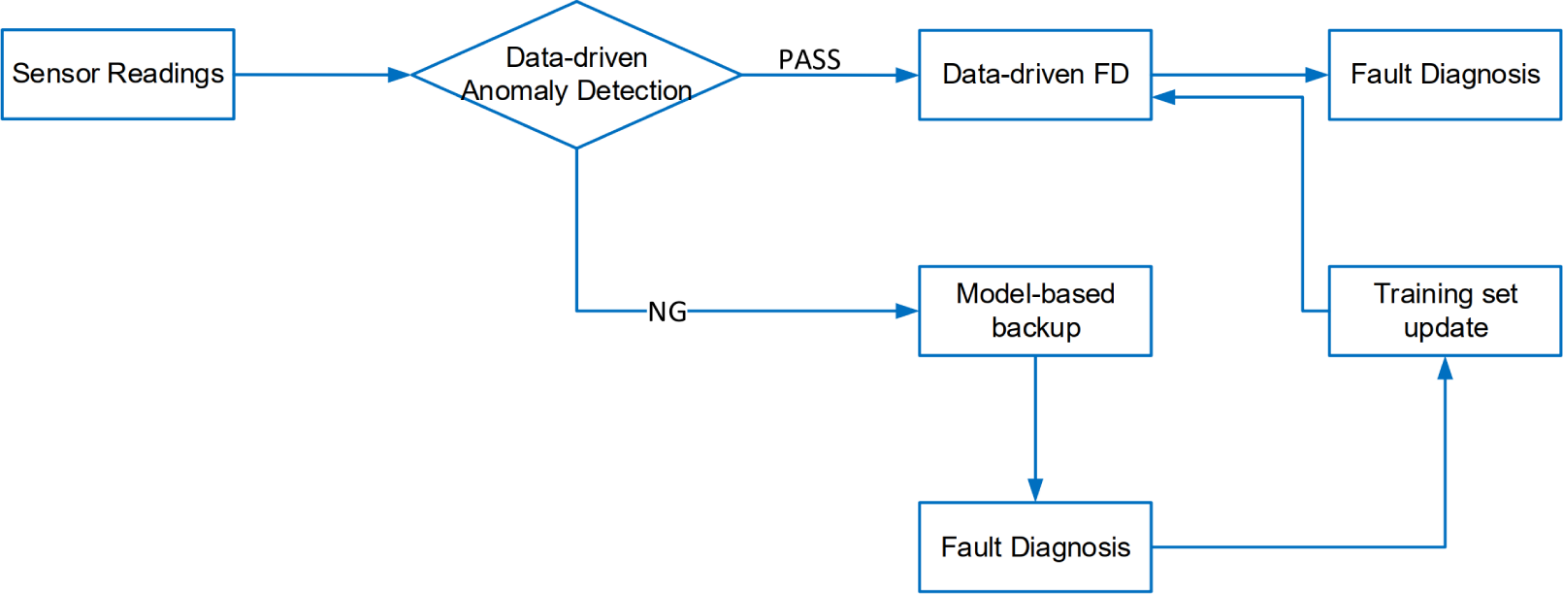
Flowchart of training set update for data-driven fault diagnosis.

This procedure is illustrated in Figure 21. Sensor readings are first fed into the anomaly detection process using the isolation forest (IF). If no anomaly is detected, the system applies the data-driven fault diagnosis as outlined in Section 3.1. However, if an anomaly is detected, a backup model-based fault diagnosis, as shown in Figure 19, is initiated. Once sufficient new data with different fault scenarios are collected, the data-driven fault diagnosis model is retrained with the updated training set to enhance its fault diagnosis capabilities under the new conditions.

### 3.3 Challenges in Data-Driven Fault Diagnosis

Data-driven methods have shown great promise in fault diagnosis due to their ability to model complex systems using historical data. However, their effectiveness is highly dependent on the quality and diversity of the training data. Without a diverse data set that includes faulty scenarios across a wide range of operational conditions, these models often fail to generalize, leading to poor performance in real-world applications, as shown in Figure 15. When properly trained with comprehensive data that captures all potential fault types and operating conditions, data-driven models excel in fault detection and diagnosis, offering accurate solutions, as shown in Figure 20. The key lies in ensuring that the training data cover all critical scenarios.

**Figure 22:**
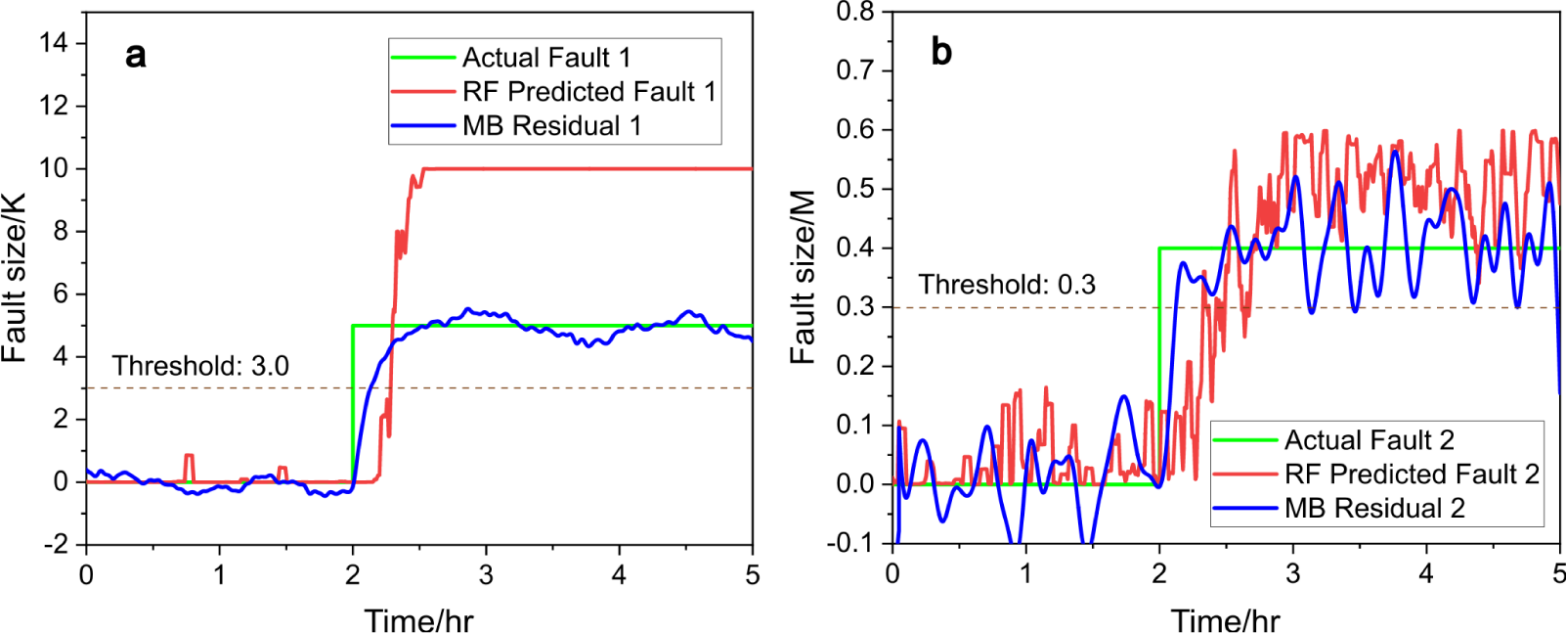
Predicted fault size vs actual fault size (green), (a) for fault 1 and (b) for fault 2 with RF (Red) and model-based residual generator (blue).

The question arises whether data are required for all fault sizes. To explore this, a test was conducted using a training set with only larger fault sizes (fault 1 at 10 and fault 2 at 0.6), while the testing set comprised smaller faults (fault 1 at 5 and fault 2 at 0.4). Figure 22 illustrates the fault diagnosis results for both fault 1 and fault 2, comparing the actual fault values, predicted fault values, and residual signals. This test provides insight into how well the models generalize to smaller fault sizes when they are trained only on larger faults.

In Figure 22b, fault 2 is introduced at the 2 h mark, with the green line indicating the actual fault size of 0.6. Prior to this, the noise in both the predicted fault signal (red line) and the residual signal (blue line) stays well below the threshold of 0.3, indicating no false positives before the fault occurs. After 2 h, both the predicted fault signal and the residual signal increase, with the red line overestimating the actual fault size of 0.4, while the blue residual signal fluctuates but stays close to the expected value of 0.4. Similar results were achieved using KNN; additionally, ANN and RNN were applied, yielding comparable results (not included in the article).

This phenomenon highlights the importance of having diverse training scenarios for accurate fault diagnosis, a requirement that may be difficult to achieve in real industrial settings. The question of what constitutes the minimal training set catches our attention. Further studies are needed to address this.

## 4 Conclusion

In this study, the effectiveness of data-driven methods such as RF and KNN for fault diagnosis in a CSTR chemical reactor system is evaluated compared to model-based residual generators. The focus is on detecting, isolating, and estimating the size of two key faults: fault 1, a coolant inlet temperature spike, and fault 2, a decrease in the 3-picoline feed concentration. Both RF and KNN demonstrated strong performance under nominal conditions, accurately identifying faults and estimating their sizes. However, both data-driven and model-based approaches faced difficulties after a change in the heat transfer coefficient (*U*), a process condition change, which led to misaligned predictions and false positives. To address this, an IF algorithm is employed for anomaly detection, allowing system model recalibration and restoring the accuracy of the model-based residual generators. For data-driven methods, the inclusion of new data sets with updated parameters in the training successfully restored their performance.

Overall, while model-based methods remain reliable due to their deep understanding of system dynamics, data-driven approaches offer scalability and efficacy without the need for detailed system models. The integration of both methods into a combined framework offers an optimal solution.

A challenge with data-driven methods is their reliance on diverse training data sets; without sufficient variety, fault diagnosis accuracy diminishes, as seen in overestimations when trained only on larger fault sizes. Future work will focus on enhancing the robustness of data-driven methods, particularly by optimizing training sets with comprehensive fault scenarios and improving their ability to handle system parameter changes to achieve fault diagnosis accuracy comparable to model-based approaches. And we will develop and test new models to enhance sensitivity and accuracy in detecting smaller faults. The sensitivity analysis of heat transfer efficiency will be conducted when sufficient data covering a broader range of *U* values becomes available. To further enhance the applicability of our approach, future work will explore strategies for reducing prediction delays, such as optimizing model architectures for efficiency or implementing online learning techniques to enable real-time performance. We will also integrate data-driven and model-based methods through adaptive model adjustment, where data-driven techniques dynamically refine model parameters, and physics-guided learning, which implants physical constraints into neural networks. Additionally, transfer learning using model-based simulations will be explored to improve data-driven models in scenarios with limited real-world fault data.

## Supporting Information Available

Design parameters of CSTR experiment in Table S1. Parameters for all data-driven methods in Table S2 and Table S4. Moving average filtering performance in terms of determination coefficient in Table S3. Average time consumption for 1 cycle of training and testing in Table S5 and the performance across scenario a to e in Table S6 (PDF)
