# Supplementary material for: Fault Diagnosis in Chemical Reactors with Data-Driven Methods
Source: Ind Eng Chem Res. 2025 Mar 8;64(11):6060–76. doi: 10.1021/acs.iecr.4c04042 (PMC11926869; doi:10.1021/acs.iecr.4c04042)
Supplement: Supplementary file 1 — ie4c04042_si_001.pdf [file ie4c04042_si_001.pdf]

Supporting Information

Fault Diagnosis in Chemical Reactors with Data-Driven Methods

Pu Du[1], *Nabil M. Abdel Jabbar*[2], *Benjamin A. Wilhite*[1] *and Costas Kravaris*[1*]

1. Artie McFerrin Department of Chemical Engineering, Texas A&M University, College Station, TX 77843

2. Chemical and Biological Engineering Department, American University of Sharjah, Sharjah 26666, United Arab Emirates

[*] Corresponding author E-mail: kravaris@tamu.edu

Property		Symbol(Control point)	Value	Unit
Total suction volumetric flow rate		F(FC4)	2.05	ml/min
3-Picoline flow rate		F_A(FC2)	1.12	ml/min
Hydrogen peroxide flow rate		F_B(FC1)	0.88	ml/min
Reactor liquid volume		V	50	ml
Coolant feed flow rate		F_j(FC3)	5	ml/min
Jacket volume		Vj	67	ml
Overall heat transfer coefficient		U	18	$W/(K\cdot m^2)$
Heat transfer surface area		A	0.08	m^2
Average density of feed		ρ	900	g/L
Average specific heat of feed		Cp	3.0	J/(g·K)
Density of coolant		ρ_j	1000	g/L
Specific heat of coolant		Cp_j	4.1	J/(g·K)
**Parameters for fault 2 into the**	3-Picoline solution feed molar conc.(Nominal)	$C_{A,in}$	2.56	mol/L
	3-Picoline conc. Under 0.4 feed ratio fault		2.16	mol/L
	3-Picoline conc. Under 0.6 feed ratio fault		1.96	mol/L
H_2O_2 solution feed molar conc.		$C_{B,in}$	5.12	mol/L
Enthalpy of the reaction		ΔH	-160	kJ/mol
Reactant feed-in temperature		T_{in}(TIC1)	340	K
**Parameters for fault 1 into the**	Coolant feed-in temperature (Nominal)	$T_{j,in}$(TIC2)	333	K
	Coolant feed-in temp. under 5 increase fault		338	K
	Coolant feed-in temp. under 10 increase fault		343	K
Catalyst conc.		Z	0.002	mol/L

Table S1: Design parameters of the CSTR (with fault introduced parameters)

**Method**	**RF**		**KNN**		**IF**	
**Parameter values**	Estimator Number	200	Neighbours Number	7	Estimator Number	200
	Maximum Depth	None	Distance Metric	Euclidean	Maximum Samples	256
	Minimum Split	2	Weights	Uniform	Maximum Features	1.0
	Minimum Leaf	2	Algorithm	Ball_tree	Contamination	0.2
	Criterion	Gini	Leaf Size	50	Random State	42
	Max Features	Sqrt				

Table S2: Parameters for data-driven methods of RF, KNN and IF

**Step points**	300	600	900	1200	1500	1800
**R^2**	0.8506	0.8813	0.9130	0.9602	0.9603	0.9677

Table S3: Moving average filtering performance in terms of determination coefficient (R^2)

**Parameter** / **Methods**	Hidden layers	Activation	Loss Function	Epoch/ Batch	Optimizer	Dropout
**ANN**	2 Dense, 64 neurons	ReLU	MSE	50/32	Adam	0.2
**RNN**	2 SimpleRNN, 64 units	ReLU	MSE	50/32	Adam	0.2

Table S4: Parameters for data-driven methods of ANN and RNN

Methods	KNN	RF	ANN	RNN
Time (s)	20	150	5100	6300

Table S5: Average time consumption for 1 circle of training and testing

R^2	Scenario a				Scenario b				Scenario c				Scenario d				Scenario e			
**Methods**	**KNN**	**RF**	**ANN**	**RNN**	**KNN**	**RF**	**ANN**	**RNN**	**KNN**	**RF**	**ANN**	**RNN**	**KNN**	**RF**	**ANN**	**RNN**	**KNN**	**RF**	**ANN**	**RNN**
**Fault 1**	0.92	0.96	0.94	0.94	NA	NA	NA	NA	0.78	0.81	0.85	0.75	0.82	0.83	0.84	0.84	0.58	0.67	0.80	0.76
**Fault 2**	NA	NA	NA	NA	0.80	0.79	0.84	0.81	0.79	0.71	0.82	0.65	0.73	0.75	0.71	0.57	0.72	0.71	0.82	0.80

Table S6: Comparison of R^2 with data-driven methods (RF, KNN, ANN and RNN) in fault size estimation
